# Single-cell analysis reveals alternations between the aged and young mice prostates

**DOI:** 10.1186/s40364-024-00666-x

**Published:** 2024-10-09

**Authors:** Yang Li, Yuhong Ding, Yaxin Hou, Lilong Liu, Zhenghao Liu, Zhipeng Yao, Pengjie Shi, Jinxu Li, Ke Chen, Junyi Hu

**Affiliations:** 1 Department of Urology, Tongji Hospital, Tongji Medical College, Huazhong University of Science and Technology, Wuhan, China; 2 Institute of Urology, Tongji Hospital, Tongji Medical College, Huazhong University of Science and Technology, Wuhan, China

**Keywords:** Aging, Prostate, ScRNAseq, Mouse

## Abstract

**Background:**

Aging of the male prostate is an inevitable process in which the prostate undergoes hyperplasia, and this growth may lead to compression of the urethra, resulting in voiding dysfunction and associated symptoms, and an increased risk of prostate cancer. Despite the significance of prostate aging, the molecular mechanisms involved are still not fully understood.

**Methods:**

Prostate split by lobes from young (2 months) and aged (24 months) mice were collected for single-cell RNA sequencing (scRNA-seq) analysis. Tissues from both anterior prostate (AP) and ventral/dorsal/lateral prostate (VDLP) were included in the study. Data analysis included unsupervised clustering using the uniform manifold approximation and projection (UMAP) algorithm to identify distinct cell types based on marker gene expression. Differential gene expression analysis was performed to identify age-related changes in gene expression across different cell types. Functional enrichment analysis was conducted to elucidate biological pathways associated with differentially expressed genes. Additionally, cellular interactions and developmental trajectories were analyzed to characterize cellular dynamics during prostate aging.

**Results:**

The single-cell transcriptome analysis of the mouse prostate during aging revealed heterogeneity across various cell types and their changes during the aging process. We found a significant increase in the proportion of mesenchymal and immune cells in aged mice. Our study unveiled alterations in genes and pathways associated with cellular senescence, oxidative stress, and regeneration in epithelial cells. Furthermore, we observed that basal cells may undergo epithelial-mesenchymal transition (EMT) to become mesenchymal cells, particularly prominent in aged mice. Additionally, immune cells, notably macrophages and T cells, exhibited a heightened inflammatory response in aged mice.

**Conclusion:**

In summary, our study provides a comparative analysis of the single-cell transcriptome of the aged and young mice prostates, elucidating cellular and molecular changes between the aged and young mice prostates.

**Supplementary Information:**

The online version contains supplementary material available at 10.1186/s40364-024-00666-x.

## Background

Aging is an inevitable and universal process affecting all cells, tissues, organs, and organisms, from unicellular organisms to mammals like mice and humans. It is governed by natural laws and entails complex biological changes intrinsic to life's progression [1, 2].

The prostate, a vital component of the male reproductive system resembling a chestnut in shape, undergoes aging starting around the age of 40 in men [3]. Unlike many organs that atrophy with age, the prostate experiences hyperplasia, leading to increased cell number and volume. Ultimately, this growth can lead to urethral compression, causing voiding dysfunction and associated symptoms, along with an elevated risk of prostate cancer [4–7].

Despite its significance, the molecular mechanisms underpinning prostate aging remain incompletely understood. Thus, a thorough understanding of prostate aging is imperative. Single-cell RNA sequencing has emerged as a potent tool for unraveling cellular heterogeneity and subtle gene expression changes during aging [8–14]. Although single-cell studies on the prostate have been abundant [15–18], they have not been systematically and deeply applied to characterize prostate aging in mice. While access to disease-free human prostate tissue is limited, model organisms like mice, which share genetic and physiological similarities with humans, offer an alternative for investigating aging mechanisms.

In this study, we employed scRNA-seq to comprehensively profile the cellular landscape of the mouse prostate and explore age-related alterations in specific cell types. By analyzing prostate tissue from young and aged mice, our goal was to elucidate dynamic changes in epithelial, stromal, and immune cell populations during aging. Our findings not only enhance our understanding of the molecular mechanisms driving prostate aging but also provide a valuable foundation for identifying potential biomarkers and developing treatments for aging-related diseases.

## Material and methods

### Animals and tissue isolation

Wild type (WT) C57BL/6 J male mice (2-months-old, 12-months-old and 24-months-old) was purchased from Beijing Vital River Laboratory Animal Technology Co., Ltd. Mouse prostates were isolated according to previously reported methods [15, 17].

### Cell dissociation

Mouse prostates were dissected and transferred to a 2 ml Eppendorf tube containing tissue storage buffer (Miltenyi Biotec, cat. no. 130–100-008). The tube was centrifuged at 50 g for 1 min at 4 °C and 1 ml preheated digestion solution ((Miltenyi Biotec, cat. no. 130–110-203), 37 °C) was added into the prostate tissue after discarding the tissue storage buffer. Tube was incubated in a 37 °C water bath and gently pipetted three times every 5 min. Enzymatic incubation time was about 15 min. When the incubation was finished, 10% FBS (Pricella, cat. no.164210–50) was added and the solution was filtered using a 40-µm cell strainer (Corning, cat. no.CLS431750). The suspensions were centrifuged at 300 g for 3 min at 4 °C, and the pellets were resuspended in PBS with 0.01% Bovine serum albumin (Sigma, cat. no. A1933).

### Single-cell RNA-seq library preparation and sequencing

Then cells were loaded into microfluidic chip of Chip A Single Cell Kit v2.0 (MobiDrop, cat. no. S050100201) to generate droplets with MobiNova-100 (MobiDrop, cat. no.A1A40001). Each cell was involved into a droplet which contained a gel bead linked with up to millions oligos (cell unique barcode). After encapsulation, droplets suffer light cut by MobiNovaSP-100 (MobiDrop, cat. no.A2A40001) while oligos diffuse into reaction mix. The mRNAs were captured by cell barcodes with cDNA amplification in droplets. Following reverse transcription, cDNAs with barcodes were amplified, and a library was constructed using the High Throughput Single Cell 3’RNA-Seq Kit v2.0 (MobiDrop, cat. no. S050200201) and the 3' Single Index Kit (MobiDrop, cat. no. S050300201). The resulting libraries were sequenced on an Illumina NovaSeq 6000 System.

### Raw data processing

MobiVision v3.0 was used to quantify raw data. All treatments were performed according to the official instructions for use, which can be referred to https://www.mobidrop.com/.

### Quality control and cell-type identification

The QC process was performed using Seurat (version 4.3.0) [19].Single cells with less than 400 UMIs or with more than 5000 UMIs or with more than 20% mitochondrion derived UMI counts were considered as low-quality cells and removed. DoubletFinder package (version2.0.3) was used for calculating doublets [20]. The mean–variance-normalized bimodality coefficient (BCMVN) of each sample was used to calculate the neighborhood size (pK), and the number of artificial doublets (pN) was set to 0.25. Batch effects among the patients were eliminated using the Harmony [21]. The top 30 principal components, along with the top 2,000 variable genes, were used in this process. Subsequently, the main cell clusters were identified using the FindClusters function (resolution = 0.8) of Seurat and visualized using 2D UMAP or tSNE [22]. Finally, 68,214 single cells, including 27,460 aged-derived cells and 40,754 young-derived cells, were subjected to further investigation.

The FindAllMarkers function was used to list the markers of each cell cluster. The major cell types were then recognized based on the markers obtained from the CellMarker database [23] and previous studies [15, 17, 18, 24].Markers used in this pipeline are addressed in Table S1. Additionally, differentially expressed genes (DEGs) of every cell type are listed in Table S2.

### DEGs analysis and enrichment analysis

DEGs were identified using the FindMarkers function of Seurat. The following cutoff threshold values were used: adj.*p*val < 0.01 and |log2Foldchange|> 1.5. DEGs between aged and young mice are listed in Table S2. Then, these DEGs were loaded into clusterProfiler [25] for the GO enrichment analysis. Pathways for which the adj.pval was < 0.05 were considered significantly enriched. MSigDB Hallmark gene sets [26] were used to compute scores using the AddModuleScore of Seurat.

### Trajectory inference and analysis

Pseudo-temporal analysis was performed with Monocle2/Monocle3 to determine the dramatic translational relationships among cell types and clusters. The following parameters were set: mean expression ≥ 0.125, num_cells_expressed ≥ 10, qval < 0.01 (differentialGeneTest function). Further detection with the Monocle2 plot_pseudotime_heatmap function revealed the key role of a series of genes in the differentiation progress [27, 28].

### Cell–cell communication analysis

Cell–cell communication analysis was conducted utilizing the CellChat (version 1.6.1) [29]. Only the expression of ligands and receptors in above 10 percent of cells from either aged or young groups were then analyzed, and only *p* value < 0.01 was retained for predicting cell–cell interaction.

### Western blot

Prostate tissue is first ground into fragments and lysed using RIPA buffer containing protease and phosphatase inhibitors. The resulting cell lysates are then centrifuged to obtain the supernatant, and protein concentration is measured using the BCA assay. The samples are mixed with loading buffer and heated to denature the proteins. Equal amounts of protein are loaded onto an SDS-PAGE gel for electrophoresis. After the electrophoresis, a wet transfer system is used to transfer the proteins from the gel onto a PVDF membrane. To block non-specific binding sites, the membrane is covered with a 5% BSA solution at room temperature for one hour, followed by a wash with TBST. The membrane is then incubated with primary antibodies on a shaker at 4 °C. After incubation, the membrane is washed three times with TBST before being treated with HRP-conjugated Anti-Mouse IgG at room temperature. Finally, after three additional TBST washes, the membrane is exposed using ECL reagent, and images are captured using a chemiluminescent imaging system.

### Multicolour immunofluorescence (IF) staining assay

An EDTA antigen retrieval buffer (pH 8.0, B0035, Powerful Biology) was applied for 15 min to facilitate antigen retrieval. To neutralize endogenous peroxidase, the samples were incubated in 3% H_2_O_2_ at room temperature for 25 min. Nonspecific binding sites were blocked by incubating the samples in 3% BSA for 30 min. The procedure involved four sequential cycles of incubation, each consisting of primary antibodies, HRP-labeled secondary antibodies, and TSA-conjugated fluorescein. After each cycle, the sections were microwaved in EDTA antigen retrieval buffer (pH 8.0) for 25 min. The following antibodies were used: CK5 (1:200,66,727–1-lg, Proteintech), CDH1 (1:200,20,874–1-AP, Proteintech), VIM (1:500,60,330–1-Ig, Proteintec h), CD3 (1:200,17,617–1-AP, Proteintech), MCP1 (1:500, GB11199, Servicebio), CD14 (1:2 00, ab182032, Abcam), CX3CR1 (1:1000, ab308613, Abcam).

### Statistical analyses

All statistical analyses and graph generation were performed in R (version 4.3.2) and GraphPad Prism (version 9.0).

## Results

Construction of single-cell transcriptome profiles of the mouse prostate during aging.

**Fig. 1:**
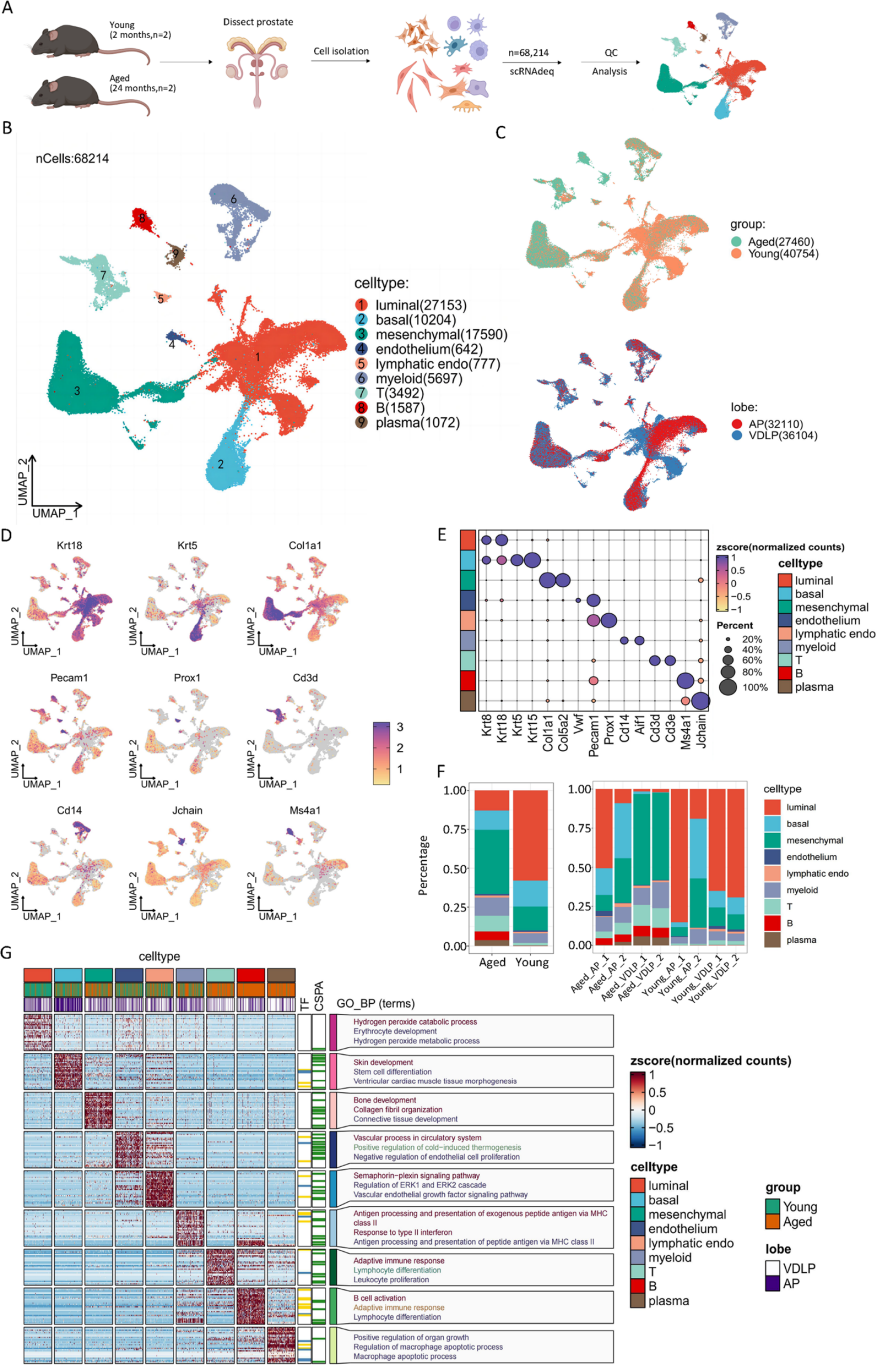
Construction of single-cell transcriptome profiles of the mouse prostate during aging. **A** Flowchart overview of the experimental design of this study. **B** UMAP plot of cells from young (2-month) and aged (24-month) mice prostates tissues grouped by cell types. **C** UMAP plot of cells from young and aged mice prostates tissues grouped by age groups(top) and lobes(bottom). **D** Feature plot of markers used to annotate major cell types. **E** Dot plot of markers used to annotate major cell types. **F** Bar plot of cell types ratios in different groups(left) and samples(right). **G** Heatmap of DEGs of every cell types and GO enriched pathways

To investigate cellular and molecular changes in gene expression during mouse prostate aging at single-cell resolution, we analyzed prostate tissues from two "young" (2 months) and two "aged" (24 months) mice using scRNA-seq (Fig. 1A). This included samples from both AP and VDLP. In total, 68,214 cells were sequenced and analyzed downstream, comprising 27,460 cells from aged mice and 40,754 cells from young mice, with 32,110 cells from AP and 36,104 cells from VDLP (Fig. 1B, C, Supplementary Fig. 1A).

We employed an unsupervised visualization approach using the UMAP algorithm, which identified nine major cell types based on the expression of typical cell type-specific markers previously reported. These cell types include luminal cells (Krt8, Krt18, *n* = 27,153), basal cells (Krt5, Krt15, *n* = 10,204), mesenchymal cells (Col1a1, Col5a2, *n* = 17,590), endothelial cells (Vwf, Pecam1, *n* = 642), lymphatic endothelial cells (Prox1, *n* = 777), myeloid cells (Cd14, Aif1, *n* = 5,697), T cells (Cd3d, Cd3e, *n* = 3,492), B cells (Ms4a1, *n* = 1,587), and plasma cells (Jchain, *n* = 1,072) (Fig. 1D,E). The cellular identities remained largely consistent during aging, with aging significantly increasing the relative proportion of mesenchymal and immune cells, while luminal cells predominated in young mice (Fig. 1F).

We further characterized these cell types by comparing cell type-specific marker genes and associated pathways in heatmaps (Fig. 1G). Gene ontology (GO) analysis of the top 30 marker genes in each cell type revealed key biological functions associated with each cell type. Overall, our study provides a comprehensive cellular landscape for single-cell transcriptome profiling of the mouse prostate during aging.

### Characterization of aging-induced changes in the molecular profile of prostate cells

**Fig. 2:**
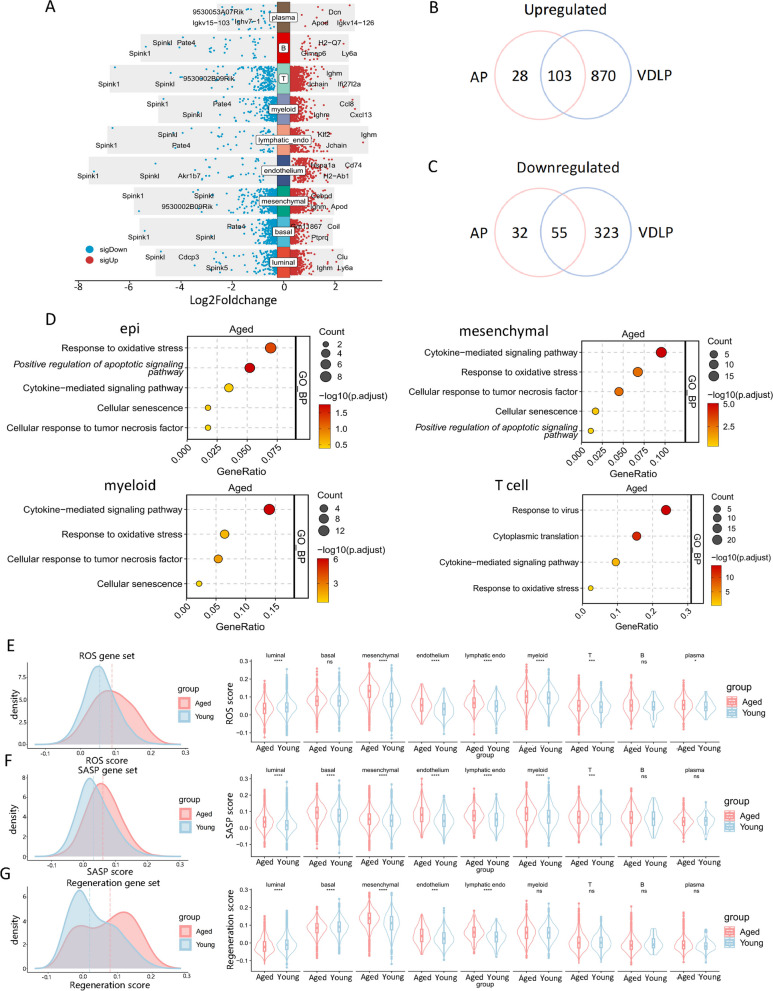
Characterization of aging-induced changes in the molecular profile of prostate cells. **A** Volcano plot of DEGs of aged and young mice of major cell types. **B** Venn plot of mutual DEGs of aged and young mice of lobes. **C** Dot plot of GO enriched pathways in epithelium, mesenchymal, myeloid, and T cells of aged mice prostates. **D** ROS gene set scores of every cell types grouped by aged and young mice. **E** SASP gene set scores of every cell types grouped by aged and young mice. **F** Regeneration gene set scores of every cell types grouped by aged and young mice

To comprehend the mechanisms underlying prostate aging, we delved into aging-induced alterations in cellular and molecular events. For the first time, we pinpointed differentially expressed genes (DEGs) in nine major cell types during mouse prostate aging, revealing multiple commonly up-regulated and down-regulated genes across different lobes of the mouse prostate (Fig. 2A-C, Supplementary Fig. 2).

Remarkably, luminal cells and B cells from aged mice exhibited high expression of Ly6a, also known as Sca1. Sca1, an 18-kDa phosphoinositide-anchoring protein and a member of the Ly-6 antigen family, serves as a cellular marker of stem cells and might be implicated in the regulation of B and T cell activation [30]. Notably, nearly all major cell types down-regulated Spink1 (Supplementary Fig. 3A), a gene encoding a protein functioning as a trypsin inhibitor, with associated pathways including the androgen receptor network in prostate cancer [31]. We downloaded prostate scRNA sequencing data of 6-month-old mice (Middle group) from public databases and compared them with our aged group, yielding similar results (Supplementary Fig. 4A, B). GO analysis of these DEGs revealed that genes up-regulated by senescence in epithelial, mesenchymal, and myeloid cells were associated with "cellular senescence," "cytokine-mediated signaling," "response to oxidative stress," and "cellular response to tumor necrosis factor." Epithelial and stromal cells were additionally associated with "positive regulation of apoptotic signaling." Genes up-regulated by senescence in T cells were associated with "response to viruses," "cytoplasmic translation," "cytokine-mediated signaling," and "response to oxidative stress" (Fig. 2). These findings indicate that the aged prostate demonstrates increased oxidative stress and cellular senescence, suggesting that aging compromises several critical metabolic processes.

We further assessed changes in several key hallmarks of aging by scrutinizing alterations in aging-related genomes in the prostate of young and aged mice. Overall gene set scores for "ROS signaling" were elevated in aged mice, notably exhibiting significantly higher scores for mesenchymal, endothelial, and immune cells, but conversely significantly lower scores for luminal cells. Intriguingly, mesenchymal and myeloid cells displayed particularly high ROS signaling scores (Fig. 2E).

Following this, we examined additional aspects of aging. Our scRNA-seq results revealed an elevated overall gene set score of SASP genes in aged mice, with significantly higher scores observed in nearly all major cell types, particularly in luminal, basal, and mesenchymal cells (Fig. 2F). Furthermore, we observed an increase in the overall gene set score of regeneration with aging. Interestingly, at the cell type level, scores were significantly decreased for both luminal and basal cells, while significantly increased for mesenchymal, endothelial, and lymphatic endothelial cells (Fig. 2G). The results were similar between the aged and middle groups (Supplementary Fig. 4G-L). These findings underscore the crucial role of various aging features, including SASP, oxidative stress, and enhanced regeneration, which exhibit cell-type-specific variations.

### Transcriptional classification of eight subpopulations of mouse prostate epithelium

**Fig. 3:**
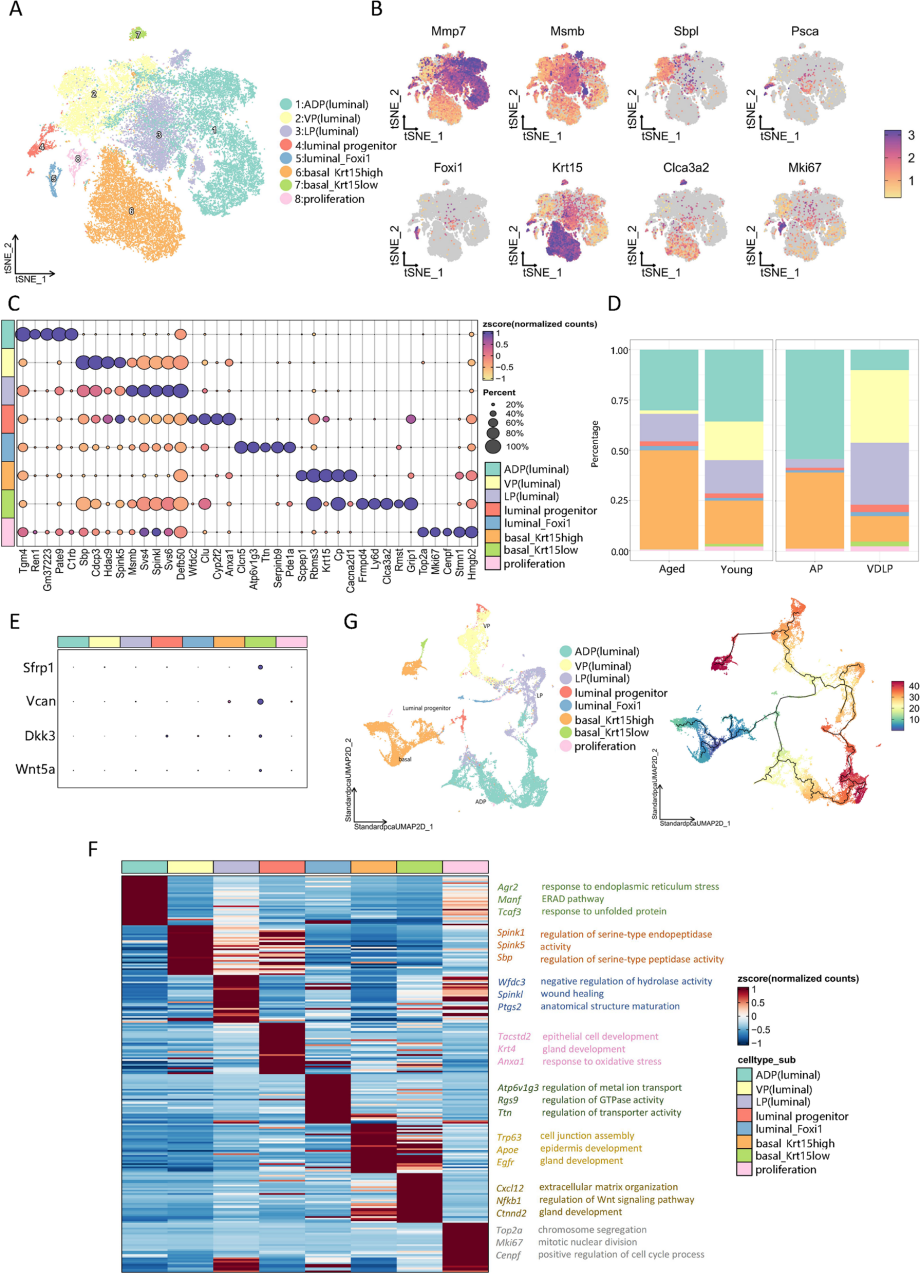
Transcriptional classification of eight subpopulations of mouse prostate epithelium. **A** tSNE plot of epitheliums grouped by cell subpopulations. **B** Feature plot of markers used to annotate epithelium subpopulations. **C** Dot plot of top 5 markers of epithelium subpopulations. **D** Bar plot of epithelium subpopulations ratios in different groups(left) and lobes(right). **E** Dot plot of Wnt related genes in epithelium subpopulations. **F** Heatmap of DEGs of every epithelium subpopulations and GO enriched pathways. **G** Dim plot of every epithelium subpopulations colored by cell types(left) and pseudotime(right)

The prostate epithelium undergoes continuous regeneration throughout life, relying on the division of luminal progenitor and basal cells to maintain homeostasis [32]. However, the impact of aging on prostate epithelial cells remains largely unclear. In this study, we investigate the heterogeneity of prostatic epithelial cells. All epithelial cells were classified into five luminal cell subsets, two basal cell subsets, and a proliferative cell subset by t-distributed Stochastic Neighbor Embedding (tSNE) analysis (Fig. 3A). Among luminal cells, lobule-specific ADP, VP, and LP epithelial cells were identified, alongside a subset of progenitor cells and a subset of intercalated cells expressing high levels of Foxi1, designated the luminal_Foxi1 subset. Basal cells were further divided into Krt15high and Krt15low subgroups based on Krt15 expression levels. Each subset exhibited a distinct transcriptional state (Fig. 3B, C).

In terms of cell proportion, aged mice exhibited increased proportions of basal_Krt15high cell subsets and decreased proportions of VP (luminal cells), basal_Krt15low, and proliferating cells, consistent with reduced regeneration scores in aged mice (Fig. 3D, Supplementary Fig. 5C). Interestingly, basal_Krt15low cells were found to secrete multiple WNT regulators such as Wnt5a and Sfrp1 (Fig. 3E). Loss of Sfrp1 is often linked to carcinogenesis in various solid tumors, suggesting that these cells may possess the ability to inhibit acinar overproliferation [33, 34]. Notably, these cells were predominantly present in young mice and exclusively in the VDLP. GO enrichment analysis indicated a potential role for these cells in gland development and regulation of the Wnt pathway (Fig. 3F).

Pseudotemporal analysis confirmed that all eight subpopulations of epithelial cells were derived from basal cells, including luminal progenitors (Fig. 3G). Basal cells differentiated into luminal progenitor cells and subsequently into epithelial cells of each lobe, including ADP (luminal cells), VP (luminal cells), and LP (luminal cells), respectively, suggesting that prostatic epithelial cells may originate from basal cells.

### Changes in gene expression of different epithelial cell subsets during prostate aging in mice

**Fig. 4:**
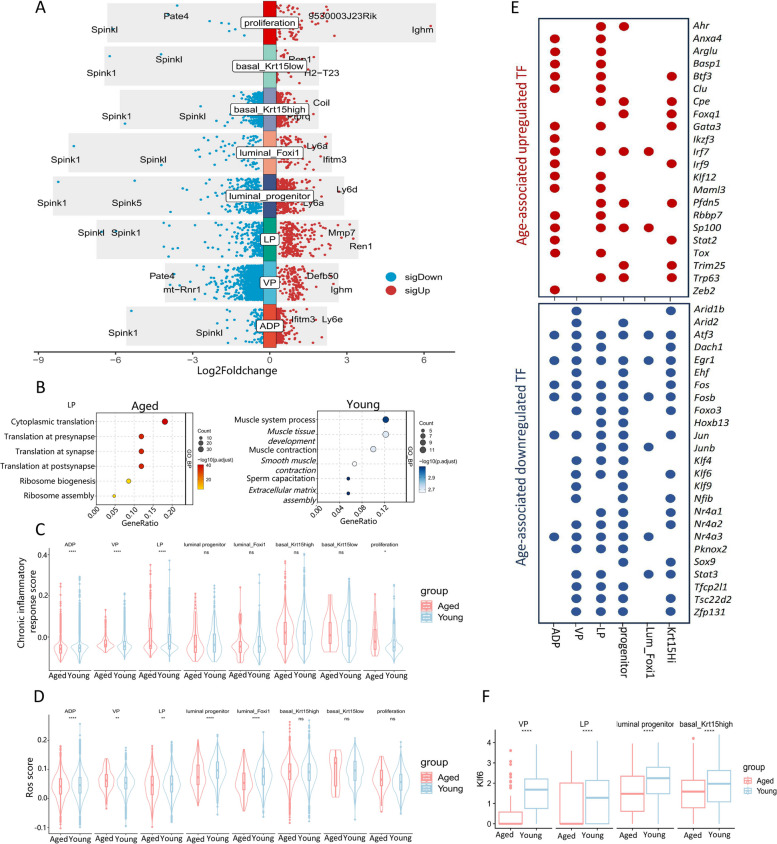
Changes in gene expression of different epithelial cell subsets during prostate aging in mice. **A** Volcano plot of DEGs of aged and young mice of every epithelium subpopulations. **B** Dot plot of GO enriched pathways in LP cells of aged(left) and young(right) mice prostates. **C** Chronic inflammatory response gene set scores of every epithelium subpopulations grouped by aged and young mice. **D** ROS gene set scores of every epithelium subpopulations grouped by aged and young mice. **E** Age-associated up/down regulated TF in epithelium subpopulations. **F** Expression of Klf6 in VP, LP, luminal progenitor, and basal_Krt15high cells grouped by aged and young mice

Next, we investigated whether epithelial cell subsets exhibited differential responses to aging. Differential analysis revealed overlapping DEGs between aged and young mice across different subsets. In aged mice, innate immune system reactive protein Ifitm3 was upregulated in ADP (luminal cells) and intercalated cell subsets, immunoglobulin heavy constant mu (Ighm) was upregulated in VP (luminal cells) and proliferation subsets, and Ly6a was upregulated in luminal progenitor cells and intercalated cell subsets [35, 36]. Consistent with major cell types, these subsets also predominantly downregulated Spink1 (Fig. 4A). GO enrichment analysis of DEGs in each subgroup indicated alterations in various pathways during aging (Fig. 4B, Supplementary Fig. 6).

We then assessed ROS and chronic inflammatory scores for each subgroup. Interestingly, chronic inflammation scores increased in VP (luminal cells), LP (luminal cells), and proliferation subsets in aged mice, while decreasing in ADP (luminal cells), with other subsets showing no significant difference. Regarding ROS scores, VP (luminal cells) scores increased in aged mice, whereas ADP (luminal cells), LP (luminal cells), luminal progenitor cells, and intercalated cells decreased. No significant difference was observed in other populations (Fig. 4C, D).

Furthermore, we conducted an analysis of transcription factors (TFs). Notably, multiple subsets exhibited shared upregulation or downregulation of TFs (Fig. 4E). For instance, ADP (luminal cells), LP (luminal cells), and basal_Krt15high all upregulated Btf3 and Gata3, while ADP (luminal cells), LP (luminal cells), progenitor cells, and intercalated cells upregulated Irf7 and Sp100. Conversely, Atf3, Egr1, and Fosb were downregulated across almost all subsets. Additionally, VP (luminal cells), LP (luminal cells), progenitor cells, and basal_Krt15high subsets showed downregulation of Klf6, which has been implicated in human epidermal aging (Fig. 4F). These findings suggest that age-related changes in key growth control TFs may play a significant role in prostate aging.

### Transcriptional classification of eight subsets of mesenchymal cells and their changes during aging

**Fig. 5:**
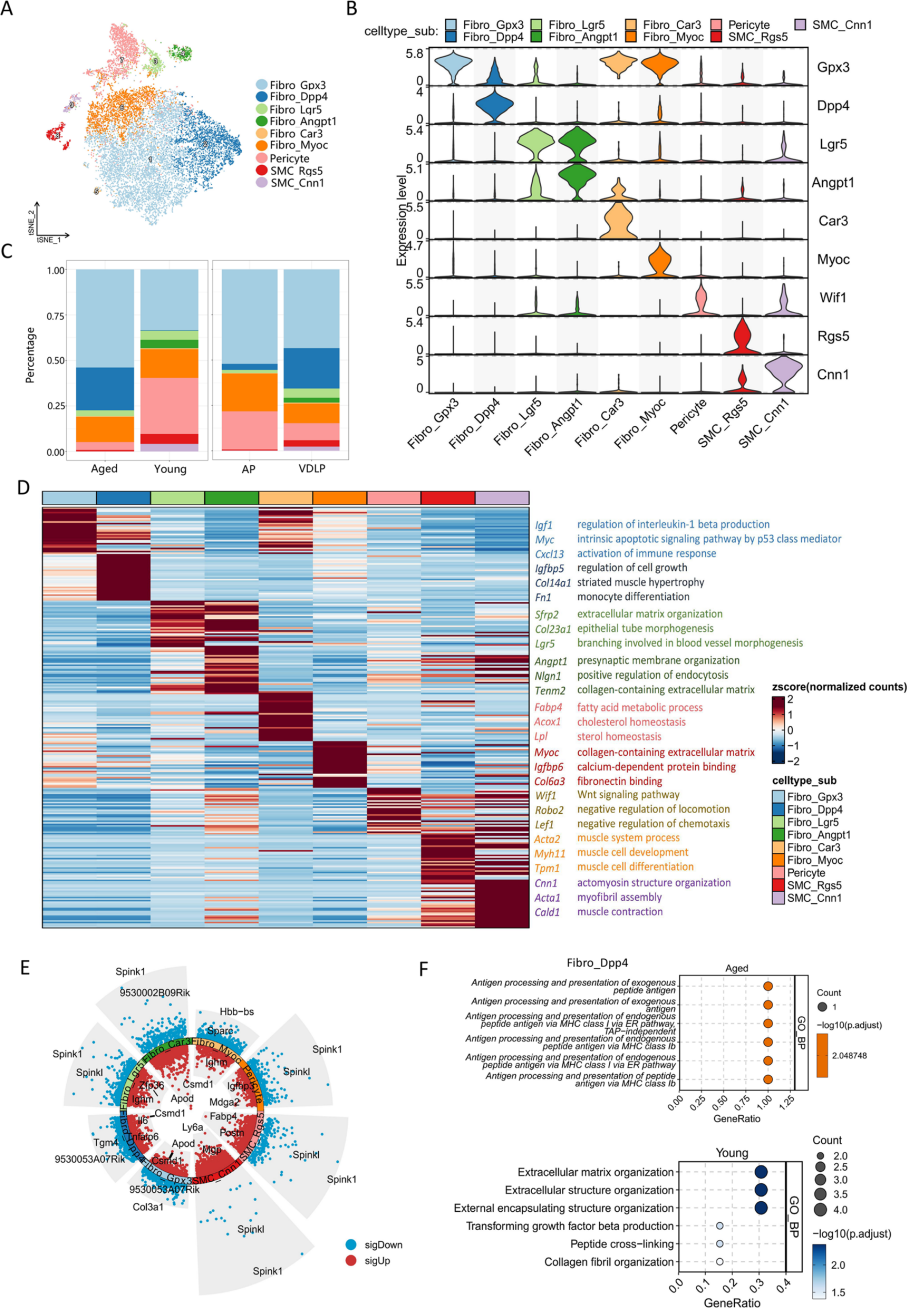
Transcriptional classification of eight subsets of mesenchymal cells and their changes during aging. **A** tSNE plot of mesenchymal cells grouped by cell subpopulations. **B** Violin plot of markers used to annotate cell subpopulations. **C** Bar plot of mesenchymal subpopulations ratios in different groups(left) and lobes(right). **D** Heatmap of DEGs of every mesenchymal subpopulations and GO enriched pathways. **E** Volcano plot of DEGs of aged and young mice of every mesenchymal subpopulations. **F** Dot plot of GO enriched pathways in Fibro_Dpp4 cells of aged(top) and young(bottom) mice prostates

The previous analysis revealed a significant increase in the proportion of mesenchymal cells in aged mice compared to young mice, yet the effects of aging on mouse prostate mesenchymal cells at single-cell resolution remain elusive. In this study, we investigate the heterogeneity of mesenchymal cells and the alterations that occur with aging. tSNE analysis classified all mesenchymal cells into six fibroblast subsets, two smooth muscle cell subsets, and one pericyte subset (Fig. 5A). Fibroblasts were denoted as Fibro_Gpx3, Fibro_Dpp4, Fibro_Lgr5, Fibro_Angpt1, Fibro_Car3, and Fibro_Myoc based on their respective marker genes, while smooth muscle cells were categorized as SMC_Rgs5 and SMC_Cnn1 subpopulations. Notably, Fibro_Dpp4 and Fibro_Myoc exhibited expression of Gpx3 to a certain extent, suggesting that Fibro_Gpx3 may represent an intermediate state between these two subsets (Fig. 5B). Interestingly, the Fibro_Dpp4 subset was predominantly present in aged mice, concomitant with an increased proportion of the Fibro_Gpx3 subset. Conversely, the Fibro_Angpt1 subset was predominantly found in young mice, accompanied by higher proportions of pericytes and smooth muscle cells (Fig. 5C, Supplementary Fig. 7A). GO enrichment analysis indicated potential roles for different subsets: Fibro_Gpx3 in "regulation of interleukin-1β production" and "p53-class mediated endogenous apoptotic signaling pathway" pathways, Fibro_Dpp4 in "regulation of cell growth" and "monocyte differentiation", Fibro_Angpt1 in "positive regulation of endocytosis", and Fibro_Myoc in "calcium-dependent protein binding" and "fibronectin binding"(Fig. 5D).

Differential analysis of these subpopulations revealed noteworthy findings. Both Fibro_Dpp4 and Fibro_Gpx3 upregulated Csmd1 in aged mice (Fig. 5E). Additionally, the Fibro_Dpp4 subset, enriched in aged mice, exhibited upregulation of several antigen presentation pathways while downregulating extracellular matrix organization and transforming growth factor beta production during aging (Fig. 5F, Supplementary Fig. 7B-K). These results suggest that mesenchymal cells in the prostatic microenvironment of aged mice undergo changes, and the unique mesenchymal cell subsets in aged mice are closely associated with the immune response.

### Basal cells may transform into mesenchymal cells through EMT

**Fig. 6:**
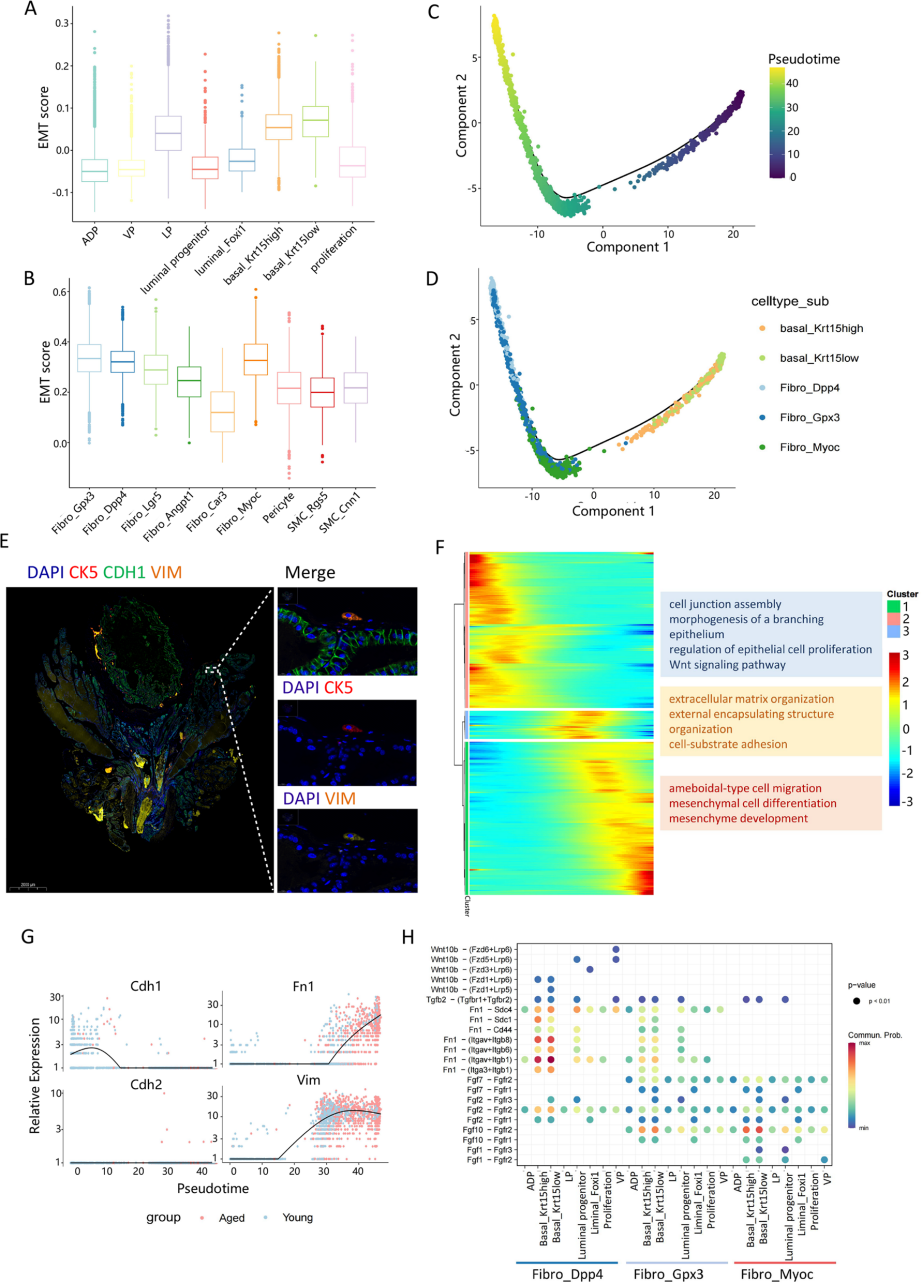
Basal cells may transform into mesenchymal cells through EMT. **A** Box plot of EMT scores grouped by epithelium subpopulations. **B** Box plot of EMT scores grouped by mesenchymal subpopulations. **C** Dim plot of basal cells, Fibro_Gpx3, Fibro_Dpp4, and Fibro_Myoc colored by pseudotime. **D** Dim plot of basal cells, Fibro_Gpx3, Fibro_Dpp4, and Fibro_Myoc colored by cell types. **E** Multicolour immunofluorescence staining of mouse prostate. **F** Heatmap of genes changed with pseudotime. **G** Expression of EMT marker genes changed with pseudotime. **H** Dot plot of mesenchymal-epithelium ligand-receptor interactions

We were intrigued by the disproportionate increase in the proportion of mesenchymal cells in aged compared to young mice. Apart from the higher chronic inflammation scores observed in LP, VP, and proliferative subsets of epithelial cells in aged mice compared to young mice, we also investigated epithelial-mesenchymal transition (EMT) scores for each epithelial and mesenchymal subpopulation. Interestingly, the two basal subsets of epithelial cells and Fibro_Gpx3, Fibro_Dpp4, and Fibro_Myoc subsets in mesenchymal cells had higher scores (Fig. 6A, B). Overall, the EMT scores of the aged group of mice were higher than those of the young group, which was validated in Western Blot (Supplementary Fig. 8A-C). Hence, we conducted a pseudo-temporal analysis of these five subpopulations, revealing divergent developmental trajectories along basal_Krt15low, basal_Krt15high, Fibro_Myoc, Fibro_Gpx3, and Fibro_Dpp4 (Fig. 6C, D). This further underscores that Fibro_Gpx3 may represent an intermediate state between Fibro_Dpp4 and Fibro_Myoc. The EMT phenotype in basal cells was validated by IF staining, which lost Cdh1 expression in favor of Vim (Fig. 6E, Supplementary Fig. 8D-F). However, we did not find this type of cells in the young mouse prostate. Remarkably, basal_Krt15low, located at the inception of the developmental trajectory, was predominantly present in young mice, while Fibro_Dpp4, situated at the terminus of the developmental trajectory, was predominantly present in aged mice. Cluster analysis of gene expression used to construct developmental trajectories delineated three clusters. Cluster 2 exhibited a gradual decrease in expression over pseudotime and was primarily associated with "branched epidermis morphogenesis" and "Wnt signaling pathway", whereas cluster 1 showed a gradual increase in expression over pseudotime and was mainly linked to "amoebic cell migration" and "mesenchymal cell differentiation" (Fig. 6E). Notably, the expression of Cdh1, whose loss is a feature of cells undergoing EMT, gradually decreased with pseudo-time, while Fn1 and Vim displayed the opposite trend [37, 38] (Fig. 6F, Supplementary Fig. 8G).

Furthermore, we investigated interactions between these three fibroblast subsets and all epithelial cells. Cell communication analysis revealed that Fibro_Dpp4 interacted with both basal cell subsets through ligand-receptor pairs such as Fn1-(Itgav + Itga1), while Fibro_Gpx3 and Fibro_Myoc interacted with both basal cell subsets through Fgf10-Fgfr2 (Fig. 6G). Fibro_Dpp4 played a predominant role in the Fn1 signaling pathway (Supplementary Fig. 8H). These findings suggest that basal cells in aged mice may undergo transition to mesenchymal cells through EMT.

### Aging-enriched macrophages may be associated with EMT

**Fig. 7:**
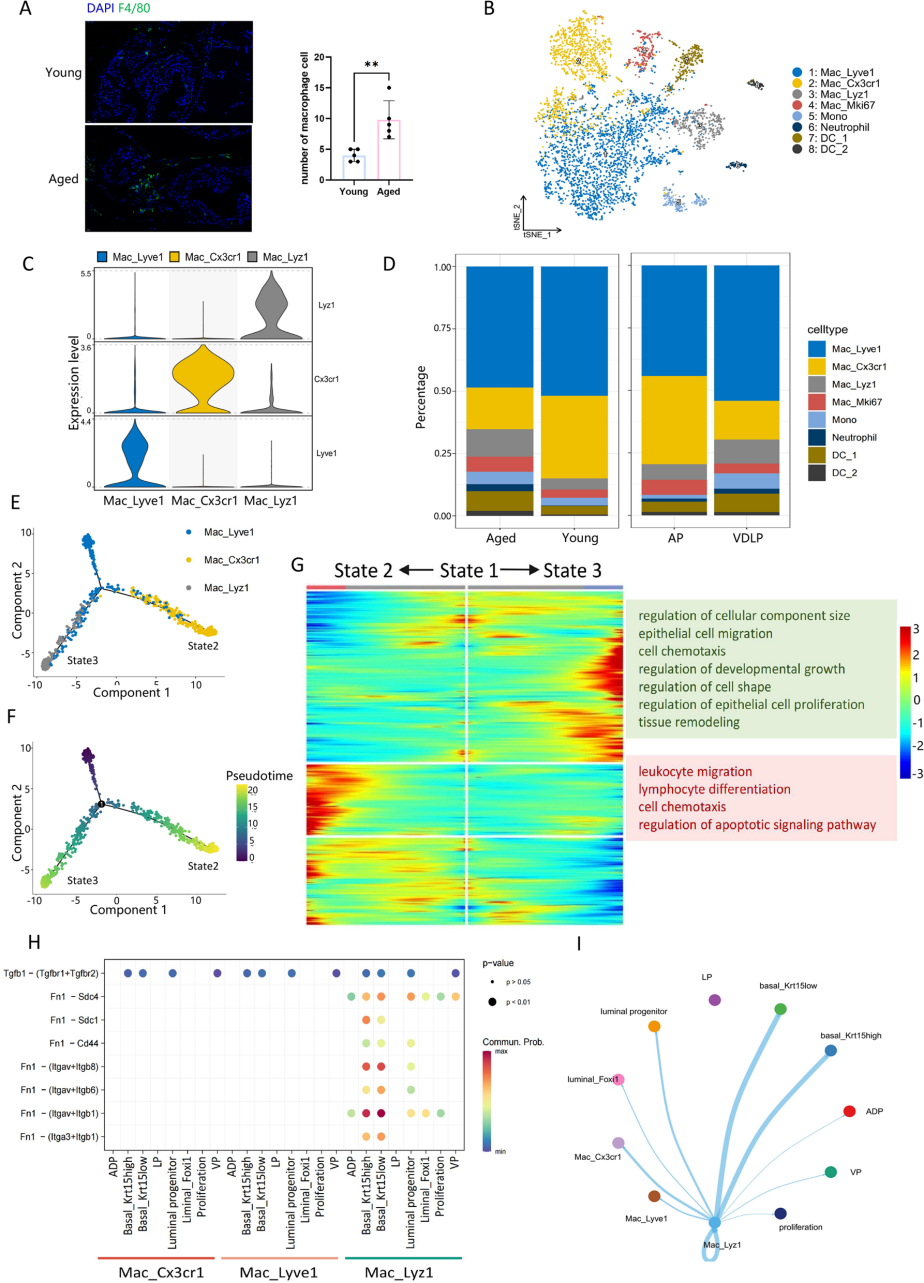
Aging-enriched macrophages may be associated with EMT. **A** Immunofluorescence staining of mouse prostate(left) and number of macrophages per 40 × magnification field(right). **B** tSNE plot of myeloid cells grouped by cell subpopulations. **C** Violin plot of markers used to annotate cell subpopulations. **D** Bar plot of myeloid subpopulations ratios in different groups(left) and lobes(right). **E** Dim plot of Mac_Lyve1, Mac_Cx3cr1 and Mac_Lyz1 colored by cell types. **F** Dim plot of Mac_Lyve1, Mac_Cx3cr1 and Mac_Lyz1 colored by pseudotime. **G** Heatmap of genes changed from State1 to State2 and State3 respectively. **H** Dot plot of macrophage-epithelium ligand-receptor interactions. **I** Circle plot of macrophage-epithelium interactions in Fn1 signaling pathway

We also noted an increased proportion of immune cells, including myeloid cells, T/B cells, and plasma cells, in aged mice compared to young mice, prompting us to delve deeper into the changes occurring in immune cells with aging. The increase of macrophages in aged mice was demonstrated by IF staining of mouse prostate (Fig. 7A, Supplementary Fig. 9A). tSNE analysis classified myeloid cells into four macrophage subsets, one monocyte subset, one neutrophil subset, and two macrophage subsets (Fig. 7B). Macrophages were labeled as Mac_Lyve1, Mac_Cx3cr1, Mac_Lyz1, and the proliferative subpopulation Mac_Mki67 based on their respective marker genes (Fig. 7C). Notably, there was no significant difference in the proportion of Mac_Lyve1 between aged and young mice; however, Mac_Cx3cr1 was higher in young mice, while Mac_Lyz1 was higher in aged mice (Fig. 7D, Supplementary Fig. 9B).

Subsequently, we conducted pseudo-temporal analyses of these three macrophage subsets. Interestingly, the developmental trajectories exhibited branching morphology from Mac_Lyve1 to Mac_Cx3cr1 and Mac_Lyz1, respectively (Fig. 7E, F). Enrichment analysis of the genes used to construct the two branches revealed that the genes in the Mac_Cx3cr1 branch were primarily associated with the "regulation of apoptosis signaling pathway", whereas the genes in the Mac_Lyz1 branch may play a role in "regulation of cell shape" and "regulation of development and growth"(Fig. 7G).

Furthermore, we examined the interactions between these three macrophage subsets and epithelial cells. Cell communication analysis revealed that, akin to fibroblasts, the aging-enriched Mac_Lyz1 subset interacted with the two basal cell subsets through ligand-receptor pairs such as Fn1-(Itgav + Itga1), with Mac_Lyz1 predominating in the Fn1 signaling pathway (Fig. 7H, I). These results suggest that Mac_Lyz1 subpopulations enriched in aged mice may be involved in EMT.

### T cells from aged mice have a stronger inflammatory response

**Fig. 8:**
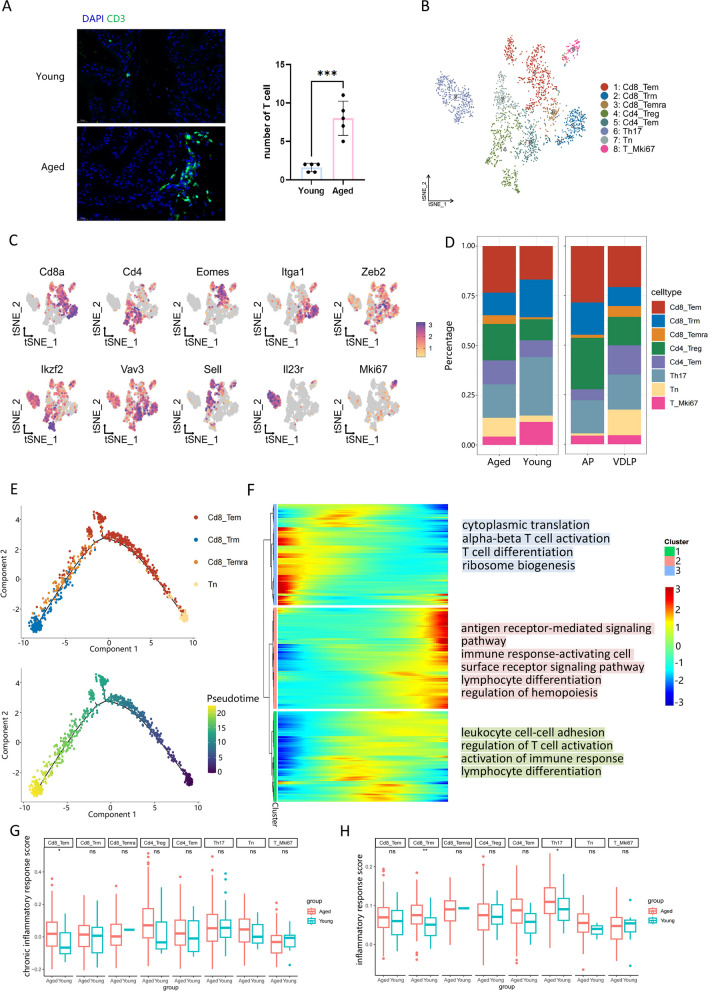
T cells from aged mice have a stronger inflammatory response. **A** Immunofluorescence staining of mouse prostate (left) and number of T cell per 40 × magnification field(right). **B** tSNE plot of T cells grouped by cell subpopulations. **C** Feature plot of markers used to annotate T cell subpopulations. **D** Bar plot of T cell subpopulations ratios in different groups(left) and lobes(right). **E** Dim plot of Tn, Cd8_Tem, Cd8_Trm and Cd8_Temra colored by cell subpopulations(top) and pseudotime(bottom). **F** Heatmap of genes changed with pseudotime. **G** Chronic inflammatory response gene set scores of every T cell subpopulations grouped by aged and young mice. **H** Inflammatory response gene set scores of every T cell subpopulations grouped by aged and young mice

The increase of T cells in aged mice was demonstrated by IF staining of mouse prostate (Fig. 8A, Supplementary Fig. 10A). We also conducted further clustering and analysis of T cells. Based on their respective marker genes, we categorized T cells into eight subsets: Cd8_Tem (effector memory), Cd8_Trm (tissue-resident memory), Cd8_Temra (terminally differentiated effector memory or effector), Cd4_Treg (regulation), Cd4_Trm, Th17, Tn (naive), and the proliferative subset T_Mki67 (Fig. 8B, C). It is noteworthy that the proportion of the Cd4_Treg subset in aged mice is higher than that in young mice, with the enriched pathways of the CD4_Treg subset primarily related to "regulation of cell–cell adhesion" and "regulation of T cell activation" (Fig. 8D, Supplementary Fig. 10B,C). Subsequently, we performed a pseudo-temporal analysis of the Cd8 and Tn subsets, revealing developmental trajectories from Tn to Tem to Temra and finally to Trm (Fig. 8E). Most of the genes utilized to map the developmental trajectories were associated with immune response (Fig. 8F).

Furthermore, we examined the scores of chronic inflammation and inflammatory response in various subsets, with the scores of aged mice generally higher than those of young mice in nearly every subgroup. Among these, the chronic inflammation score of Cd8_Tem and the inflammatory response score of Cd8_Trm and Th17 were statistically significant (Fig. 8G, H). In summary, T cells from aged mice exhibited a more pronounced inflammatory response.

## Discussion

Our study sheds light on the complex cellular and molecular landscape of the mouse prostate during aging, providing valuable insights into the mechanisms underlying age-related changes in prostate biology. The characterization of different cell populations, including epithelial, stromal, and immune cells, provides a basis for understanding the complex interactions between different cell types in the aging prostate.

It is well known that luminal cells are the most important cell type in the prostate, basal cells support and protect epithelial cells, mesenchymal cells are also widely distributed in the prostate and regulate the discharge of prostatic fluid, and immune cells play a role in defense against infection and inflammation [39]. Here, scRNA-seq results not only highlight several key processes but also reveal the heterogeneity of different cell types during prostate aging. For example, previous studies have shown that ROS is strongly associated with prostate disease, but our study showed that interstitial cells, endothelial cells and immune cells in aged mice have higher ROS scores, while luminal cells have lower scores [40, 41]. These results demonstrate cell type-specific changes in certain aging markers of prostate aging. We confirmed at the single-cell level that senescent prostates show increased oxidative stress and cellular senescence, suggesting that aging impairs several key metabolic processes. These results also suggest that oxidative stress may serve as one of the biomarkers of prostate aging and may serve as a potential diagnostic or therapeutic target for aging-related benign prostatic hyperplasia (BPH) and prostate cancer.

Furthermore, our study identified some genes that were generally downregulated or upregulated in aging prostate, such as Ly6a and Spink1. Ly6a plays an important role in the self-renewal and differentiation of stem cells. It is involved in maintaining the undifferentiated state of stem cells, and also plays a regulatory role in the differentiation process of cells [42]. Its expression in immune cells is related to the regulation of immune response. In contrast, we found that the regenerative capacity of old prostate cells was increased, which may be related to prostatic hyperplasia. SPINK1-overexpression is a molecular subtype of prostate cancer and is closely related to cancer progression and poor prognosis [43–45]. Interestingly, Spink1 was found to be significantly down-regulated in almost all cell types. This suggests that Spink1 downregulation may be related to BPH, or it may play a two-way role in the development and progression of prostate cancer.

Another key finding of our study is that mesenchymal cells, especially fibroblast subsets, are significantly increased in aged mice compared to young mice. This expansion of mesenchymal cells may reflect changes in the prostate microenvironment during aging, possibly contributing to age-related pathologies such as fibrosis and inflammation [41]. The identification of distinct fibroblast subsets with unique gene expression profiles underscores the heterogeneity of senescent prostate stromal cells and suggests different roles for stromal cells in tissue homeostasis and disease progression. In aged mice, both Fibro_Dpp4 and Fibro_Gpx3 up-regulate Csmd1, and the mutation and abnormal expression of CSMD1 have been associated with a variety of tumors, including breast cancer, esophageal cancer, colorectal cancer, and lung cancer [46–49]. Studies have shown that functional loss of CSMD1 may lead to increased cell proliferation, migration, and invasion, thereby promoting tumor development and metastasis [50, 51]. The Fibro_Dpp4 subset showed upregulation of several antigen presentation pathways during aging while downregulating extracellular matrix organization and transforming growth factor beta production, and the TGFβ1/EMT pathway is closely related to prostate cancer metastasis [37, 38].This suggests to us that CSMD1 may be a potential target worth investigating in prostate diseases.

In addition, our analysis revealed age-related changes in epithelial cell populations, including changes in basal and luminal cell subsets. We were surprised to observe “LP-like” luminal cells in the aged AP (Supplementary Fig. 5C). There may be progenitor cell activation in aged mice, leading to lineage changes in different regions of the prostate. We found that basal_Krt15low cell subset is enriched in young mice, and this subset secrets multiple WNT regulators, such as Wnt5a and strp1, the loss of which is commonly associated with the carcinogenesis of various solid tumors, suggesting that these cells may have the ability to inhibit acinar overproliferation [33, 34]. The observed decrease in proliferating cells and upregulation of genes associated with aging and inflammation in aged mice highlight the effects of aging on epithelial cell dynamics and function, and that age-related changes in key growth control TFs may play an important role in prostate aging [11, 12]. For example, Klf6 is downregulated in VP (luminal cells), LP (luminal cells) and luminal progenitor cells, which is associated with human epidermal aging.

EMT is a complex and multi-level biological process, which plays an important role in normal physiological and pathological conditions. During the repair process after tissue injury, EMT contributes to the generation of new interstitial cells that are able to migrate to the site of injury and participate in tissue reconstruction. In cancer development, EMT is considered to be one of the important mechanisms by which tumor cells acquire the ability to invade and migrate [52, 53]. Previous studies have shown that EMT plays a central role in BPH [54]. Here, we found that the conversion of basal cells to mesenchymal cells through EMT may represent a novel mechanism of prostate aging with implications for tissue remodeling and disease development.

Interestingly, our study elucidated age-related changes in immune cell populations, with an increased ratio of myeloid cells, T/B cells, and plasma cells in aged mice. Previous studies have shown that macrophages are able to restore proliferation and gene expression in epithelial and stromal cells of benign prostatic hyperplasia [55]. We identified a macrophage subset, Mac_Lyz1, which may promote EMT through cell communication, which may be involved in the development of BPH or prostate cancer. The elevated inflammatory response observed in T cells from aged mice suggests a dysregulated immune function during aging that may contribute to chronic inflammation and tissue damage in the aged prostate.

BPH and prostate cancer are two of the most common diseases affecting elderly men. Our research highlights the potential link between aging and tumor formation, suggesting that they may share a similar microenvironment, characterized by factors such as increased macrophage activity, mesenchymal cell proliferation, and ROS expression. However, we can note that ROS scores are heterogeneous between cell types, although overall the aged group scores are higher than the young group, by cell type, the scores of luminal and basal cells are decreased, while the scores of mesenchymal cells are elevated. This suggests that heterogeneity in the state of different cell types may contribute to different disease paths. We know that prostate cancer is derived from epithelial cells rather than mesenchymal cells, and perhaps if the luminal cell score is also elevated, individuals may be more likely to develop prostate cancer.

Together, our findings provide new insights into the cellular and molecular mechanisms driving prostate aging and lay the foundation for elucidating the role of specific cell populations and signaling pathways in age-related prostate disease. Although there are significant differences between mouse and human prostate, access to disease-free human prostate tissue is rather limited, especially in young samples, and mice provide an alternative option for studying the mechanisms of aging. Clinicians can use the resource data we provide to conduct more personalized analysis and search for potential therapeutic targets. Understanding the complex interplay between different cell types in the aging prostate may ultimately lead to the development of targeted therapies for age-related prostate diseases, benefiting millions of older adults worldwide.

## Conclusion

In conclusion, our study provides a comparative analysis of the aged and young mice prostates at single-cell resolution. We identified distinct cell populations and delineated age-related alterations in gene expression, cellular composition, and functional pathways. Our findings highlight the dynamic nature of the prostate microenvironment during aging, with implications for understanding age-related prostate pathologies. By elucidating the heterogeneity of cell populations and their interactions, our study contributes to a better understanding of the complex processes underlying prostate aging. Future studies focusing on the functional consequences of these age-related changes may pave the way for the development of novel therapeutic strategies for age-related prostate diseases.

## Supplementary Information

Supplementary Material 1.

Supplementary Material 2.

Supplementary Material 3.

## Data Availability

The raw sequence data reported in this paper have been deposited in the Genome Sequence Archive (Genomics, Proteomics & Bioinformatics 2021) in National Genomics Data Center (Nucleic Acids Res 2022), China National Center for Bioinformation / Beijing Institute of Genomics, Chinese Academy of Sciences (GSA: CRA018609) that are publicly accessible at https://ngdc.cncb.ac.cn/gsa [56, 57]. The data of Middle group was downloaded from Gene Expression Omnibus (GEO) database (GSE165741). No custom code or software were generated in this work. Code used in this work has been uploaded to GitHub available at https://github.com/connotations/prostate.

## Abbreviations

scRNAseq: Single cell RNA sequencing
AP: Anterior prostate
VDLP: Ventral/dorsal/lateral prostate
UMAP: Uniform manifold approximation and projection
EMT: Epithelial-mesenchymal transition
WT: Wild type
DEGs: Differentially expressed genes
GO: Gene ontology
tSNE: T-distributed Stochastic Neighbor Embedding
TFs: Transcription factors
OSCCs: Oropharyngeal squamous cell carcinomas
ROS: Reactive oxygen species
SASP: Senescence-associated secretory phenotype
BPH: Benign prostatic hyperplasia
Krt8: Keratin 8
Krt18: Keratin 18
Krt5: Keratin 5
Krt15: Keratin 15
Col1a1: Collagen Type I Alpha 1 Chain
Col5a2: Collagen Type V Alpha 2 Chain
Vwf: Von Willebrand Factor
Pecam1: Platelet And Endothelial Cell Adhesion Molecule 1
Prox1: Prospero Homeobox 1
Aif1: Allograft Inflammatory Factor 1
Ms4a1: Membrane Spanning 4-Domains A1
Jchain: Joining Chain Of Multimeric IgA And IgM
Ly6a: Lymphocyte Antigen 6 Family Member A
Spink1: Serine Peptidase Inhibitor Kazal Type 1
Btf3: Basic Transcription Factor 3
Gata3: GATA Binding Protein 3
Irf7: Interferon Regulatory Factor 7
Atf3: Activating Transcription Factor 3
Egr1: Early Growth Response 1
Klf6: KLF Transcription Factor 6
Gpx3: Glutathione Peroxidase 3
Dpp4: Dipeptidyl Peptidase 4
Lgr5: Leucine Rich Repeat Containing G Protein-Coupled Receptor 5
Angpt1: Angiopoietin 1
Car3: Carbonic Anhydrase 3
Myoc: Myocilin
Rgs5: Regulator Of G Protein Signaling 5
Cnn1: Calponin 1
Itgav: Integrin Subunit Alpha V
Itga1: Integrin Subunit Alpha 1
Fgf10: Fibroblast Growth Factor 10
Fgfr2: Fibroblast Growth Factor Receptor 2
Lyve1: Lymphatic Vessel Endothelial Hyaluronan Receptor 1
Cx3cr1: C-X3-C Motif Chemokine Receptor 1
Lyz1: Lysozyme 1
Mki67: Marker Of Proliferation Ki-67
Csmd1: CUB And Sushi Multiple Domains 1
Wnt5a: Wnt Family Member 5A
Sfrp1: Secreted Frizzled Related Protein 1
Cdh1: Cadherin 1
Fn1: Fibronectin 1
Vim: Vimentin
