# Supplementary material for: Single-cell analysis reveals alternations between the aged and young mice prostates
Source: Biomark Res. 2024 Oct 9;12:117. doi: 10.1186/s40364-024-00666-x (PMC11462726; doi:10.1186/s40364-024-00666-x)
Supplement: Supplementary file 1 — Supplementary Material 1. [file 40364_2024_666_MOESM1_ESM.pdf]

Supplementary Figure Legends

Supplementary Figure 1
(A)UMAP plot of all cells splitted by lobes and colored by age groups.

Supplementary Figure 2
(A)Volcano plot of DEGs between AP_Aged and AP_Young.
(B)Volcano plot of DEGs between VDLP_Aged and VDLP_Young.
(C,E,G,I) Bar plot of GO enriched pathways up or down regulated in luminal cells(C),basal cells(E),mesenchymal cells(G) and myeloid cells(I) between AP_Aged and AP_Young.
(D,F,H,J) Bar plot of GO enriched pathways up or down regulated in luminal cells(C),basal cells(E),mesenchymal cells(G) and myeloid cells(I) between VDLP_Aged and VDLP_Young.

Supplementary Figure 3
(A)Feature plot of Spink1 splitted by age groups.
(B-E)Dot plot of GO enriched pathways in epithelium(B), mesenchymal cells(C), myeloid cells(D), and T cells(E) of young mice prostates.

Supplementary Figure 4
(A)UMAP plot of cells from middle (6-month) and aged (24-month) mice prostates.
(B)Volcano plot of DEGs between aged and middle mice.
(C-F)Bar plot of GO enriched pathways up or down regulated in luminal cells(C),basal cells(D),mesenchymal cells(E) and myeloid cells(F) between aged and middle mice.
(G,H)ROS gene set scores of every cell types grouped by aged and middle mice.
(I,J)SASP gene set scores of every cell types grouped by aged and middle mice
(K,L)Regeneration gene set scores of every cell types grouped by aged and middle mice.

Supplementary Figure 5
(A)Dim plot of ADP(luminal cells) grouped by lobes.
(B)Dim plot of luminal cells obtained from GSE165741.
(C)Bar plot of epithelial cell subsets ratio grouped by AP_Aged vs AP_Young and VDLP_Aged vs VDLP_Young.

Supplementary Figure 6
(A,B)Dot plot of GO enriched pathways in ADP(luminal cells) of aged and young mice prostates.
(C,D)Dot plot of GO enriched pathways in basal_Krt15high cells of aged and young mice prostates.
(E,F)Dot plot of GO enriched pathways in luminal_Foxi1cells of aged and young mice prostates.

(G,H)Dot plot of GO enriched pathways in luminal progenitor cells of aged and young mice prostates.
(I,J)Dot plot of GO enriched pathways in proliferation cells of aged and young mice prostates.
(K,L)Dot plot of GO enriched pathways in VP(luminal cells) of aged and young mice prostates.

Supplementary Figure 7
(A)Bar plot of mesenchymal cell subsets ratio grouped by AP_Aged vs AP_Young and VDLP_Aged vs VDLP_Young.
(B,C)Dot plot of GO enriched pathways in Fibro_Gpx3 cells of aged and young mice prostates.
(D,E)Dot plot of GO enriched pathways in Fibro_Lgr5 cells of aged and young mice prostates.
(F,G)Dot plot of GO enriched pathways in Fibro_Myoc cells of aged and young mice prostates.
(H,I)Dot plot of GO enriched pathways in pericyte cells of aged and young mice prostates.

Supplementary Figure 8
(A)Violin plot of EMT score grouped by aged and young mice.
(B)Violin plot of Cdh1 and Vim grouped by aged and young mice.
(C)Western blot of EMT markers quantified in aged and young mice prostates.
(D-F)Multicolour immunofluorescence staining of mouse prostate verified the EMT phenotype of basal cell in VP (D), DP (E) and LP (F) lobe.
(G)Expression of EMT marker genes changed with pseudotime.
(H)Circle plot of mesenchymal-epithelium interactions in Fn1 signaling pathway.

Supplementary Figure 9
(A)Immunofluorescence staining of mouse prostate(left) and number of macrophages per 40x magnification field(right).
(B)Bar plot of myeloid cell subsets ratio grouped by AP_Aged vs AP_Young and VDLP_Aged vs VDLP_Young.
(C,D)Dot plot of GO enriched pathways in Mac_Cx3cr1 cells of aged and young mice prostates.
(E,F)Dot plot of GO enriched pathways in Mac_Lyve1 cells of aged and young mice prostates.
(G,H)Dot plot of GO enriched pathways in Mac_Lyz1 cells of aged and young mice prostates.
(I,J)Dot plot of GO enriched pathways in monocytes of aged and young mice prostates.

Supplementary Figure 10
(A)Immunofluorescence staining of mouse prostate (left) and number of T cell per

40x magnification field(right).

(B)Bar plot of T cell subsets ratio grouped by AP_Aged vs AP_Young and VDLP_Aged vs VDLP_Young.

(C)Dot plot of GO enriched pathways in T cell subpopulations.

SFig1

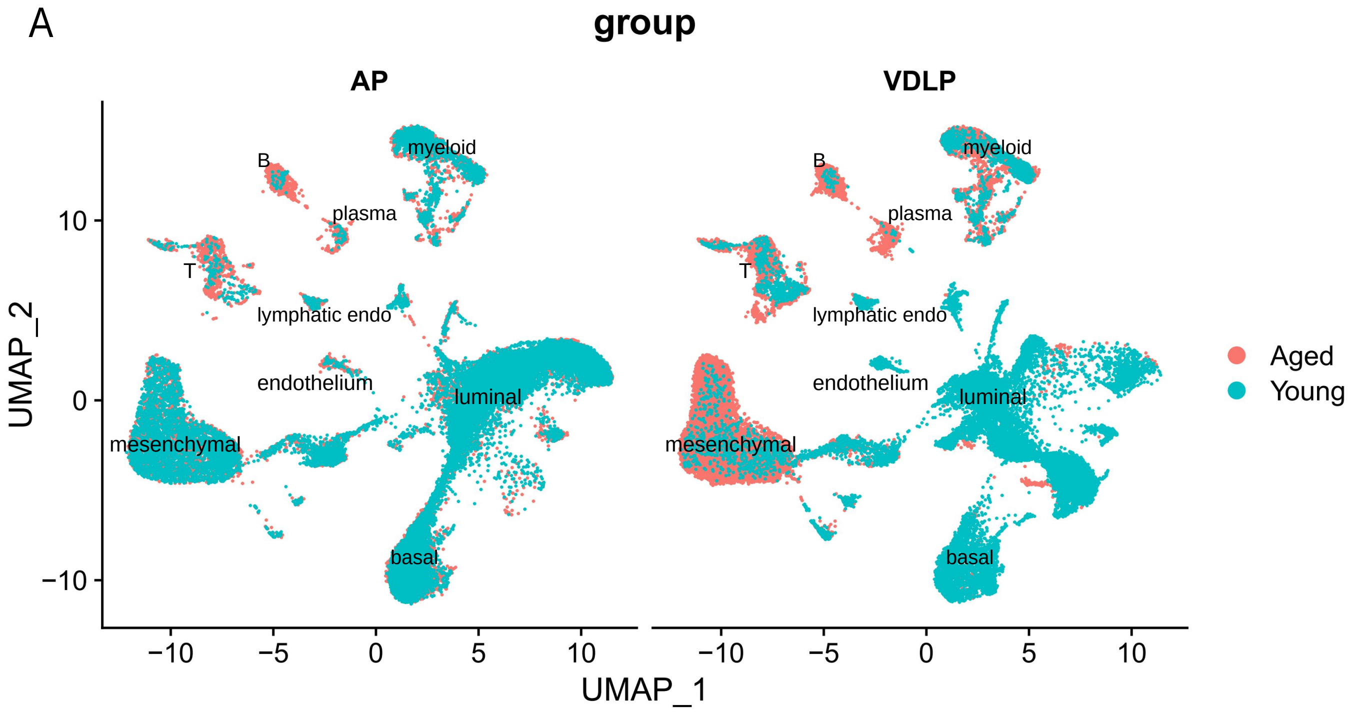
A
group
AP
VDLP
UMAP_2
UMAP_1
10
0
−10
−10
−5
0
5
10
myeloid
B
plasma
T
lymphatic endo
endothelium
luminal
mesenchymal
basal
Aged
Young

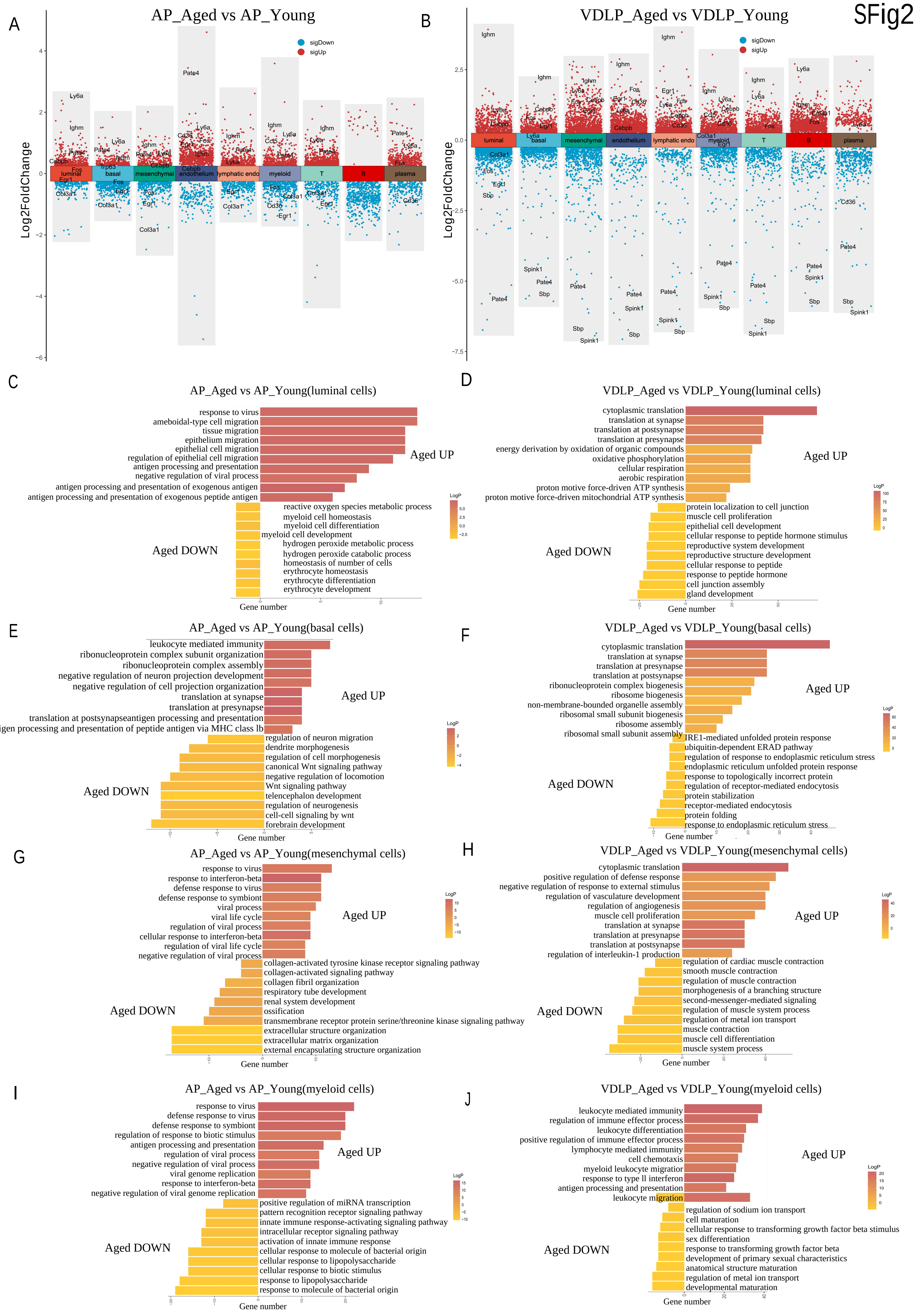

SFig2
A
AP_Aged vs AP_Young
sigDown
sigUp
Log2FoldChange
luminal
basal
mesenchymal
endothelium
lymphatic endo
myeloid
T
B
plasma
B
VDLP_Aged vs VDLP_Young
sigDown
sigUp
Log2FoldChange
C
AP_Aged vs AP_Young(luminal cells)
response to virus
ameboidal-type cell migration
tissue migration
epithelium migration
epithelial cell migration
regulation of epithelial cell migration
antigen processing and presentation
negative regulation of viral process
antigen processing and presentation of exogenous antigen
antigen processing and presentation of exogenous peptide antigen
Aged UP
reactive oxygen species metabolic process
myeloid cell homeostasis
myeloid cell differentiation
myeloid cell development
hydrogen peroxide metabolic process
hydrogen peroxide catabolic process
homeostasis of number of cells
erythrocyte homeostasis
erythrocyte differentiation
erythrocyte development
Aged DOWN
LogP
Gene number
D
VDLP_Aged vs VDLP_Young(luminal cells)
cytoplasmic translation
translation at synapse
translation at postsynapse
translation at presynapse
energy derivation by oxidation of organic compounds
oxidative phosphorylation
cellular respiration
aerobic respiration
proton motive force-driven ATP synthesis
proton motive force-driven mitochondrial ATP synthesis
Aged UP
protein localization to cell junction
muscle cell proliferation
epithelial cell development
cellular response to peptide hormone stimulus
reproductive system development
reproductive structure development
cellular response to peptide
response to peptide hormone
cell junction assembly
gland development
Aged DOWN
LogP
Gene number
E
AP_Aged vs AP_Young(basal cells)
leukocyte mediated immunity
ribonucleoprotein complex subunit organization
ribonucleoprotein complex assembly
negative regulation of neuron projection development
negative regulation of cell projection organization
translation at synapse
translation at presynapse
translation at postsynapseantigen processing and presentation
antigen processing and presentation of peptide antigen via MHC class Ib
Aged UP
regulation of neuron migration
dendrite morphogenesis
regulation of cell morphogenesis
canonical Wnt signaling pathway
negative regulation of locomotion
Wnt signaling pathway
telencephalon development
regulation of neurogenesis
cell-cell signaling by wnt
forebrain development
Aged DOWN
LogP
Gene number
F
VDLP_Aged vs VDLP_Young(basal cells)
cytoplasmic translation
translation at synapse
translation at presynapse
translation at postsynapse
ribonucleoprotein complex biogenesis
ribosome biogenesis
non-membrane-bounded organelle assembly
ribosomal small subunit biogenesis
ribosome assembly
ribosomal small subunit assembly
Aged UP
IRE1-mediated unfolded protein response
ubiquitin-dependent ERAD pathway
regulation of response to endoplasmic reticulum stress
endoplasmic reticulum unfolded protein response
response to topologically incorrect protein
regulation of receptor-mediated endocytosis
protein stabilization
receptor-mediated endocytosis
protein folding
response to endoplasmic reticulum stress
Aged DOWN
LogP
Gene number
G
AP_Aged vs AP_Young(mesenchymal cells)
response to virus
response to interferon-beta
defense response to virus
defense response to symbiont
viral process
viral life cycle
regulation of viral process
cellular response to interferon-beta
regulation of viral life cycle
negative regulation of viral process
Aged UP
collagen-activated tyrosine kinase receptor signaling pathway
collagen-activated signaling pathway
collagen fibril organization
respiratory tube development
renal system development
ossification
transmembrane receptor protein serine/threonine kinase signaling pathway
extracellular structure organization
extracellular matrix organization
external encapsulating structure organization
Aged DOWN
LogP
Gene number
H
VDLP_Aged vs VDLP_Young(mesenchymal cells)
cytoplasmic translation
positive regulation of defense response
negative regulation of response to external stimulus
regulation of vasculature development
regulation of angiogenesis
muscle cell proliferation
translation at synapse
translation at presynapse
translation at postsynapse
regulation of interleukin-1 production
Aged UP
regulation of cardiac muscle contraction
smooth muscle contraction
regulation of muscle contraction
morphogenesis of a branching structure
second-messenger-mediated signaling
regulation of muscle system process
regulation of metal ion transport
muscle contraction
muscle cell differentiation
muscle system process
Aged DOWN
LogP
Gene number
I
AP_Aged vs AP_Young(myeloid cells)
response to virus
defense response to virus
defense response to symbiont
regulation of response to biotic stimulus
antigen processing and presentation
regulation of viral process
negative regulation of viral process
viral genome replication
response to interferon-beta
negative regulation of viral genome replication
Aged UP
positive regulation of miRNA transcription
pattern recognition receptor signaling pathway
innate immune response-activating signaling pathway
intracellular receptor signaling pathway
activation of innate immune response
cellular response to molecule of bacterial origin
cellular response to lipopolysaccharide
cellular response to biotic stimulus
response to lipopolysaccharide
response to molecule of bacterial origin
Aged DOWN
LogP
Gene number
J
VDLP_Aged vs VDLP_Young(myeloid cells)
leukocyte mediated immunity
regulation of immune effector process
leukocyte differentiation
positive regulation of immune effector process
lymphocyte mediated immunity
cell chemotaxis
myeloid leukocyte migration
response to type II interferon
antigen processing and presentation
leukocyte migration
Aged UP
regulation of sodium ion transport
cell maturation
cellular response to transforming growth factor beta stimulus
sex differentiation
response to transforming growth factor beta
development of primary sexual characteristics
anatomical structure maturation
regulation of metal ion transport
developmental maturation
Aged DOWN
LogP
Gene number

SFig3

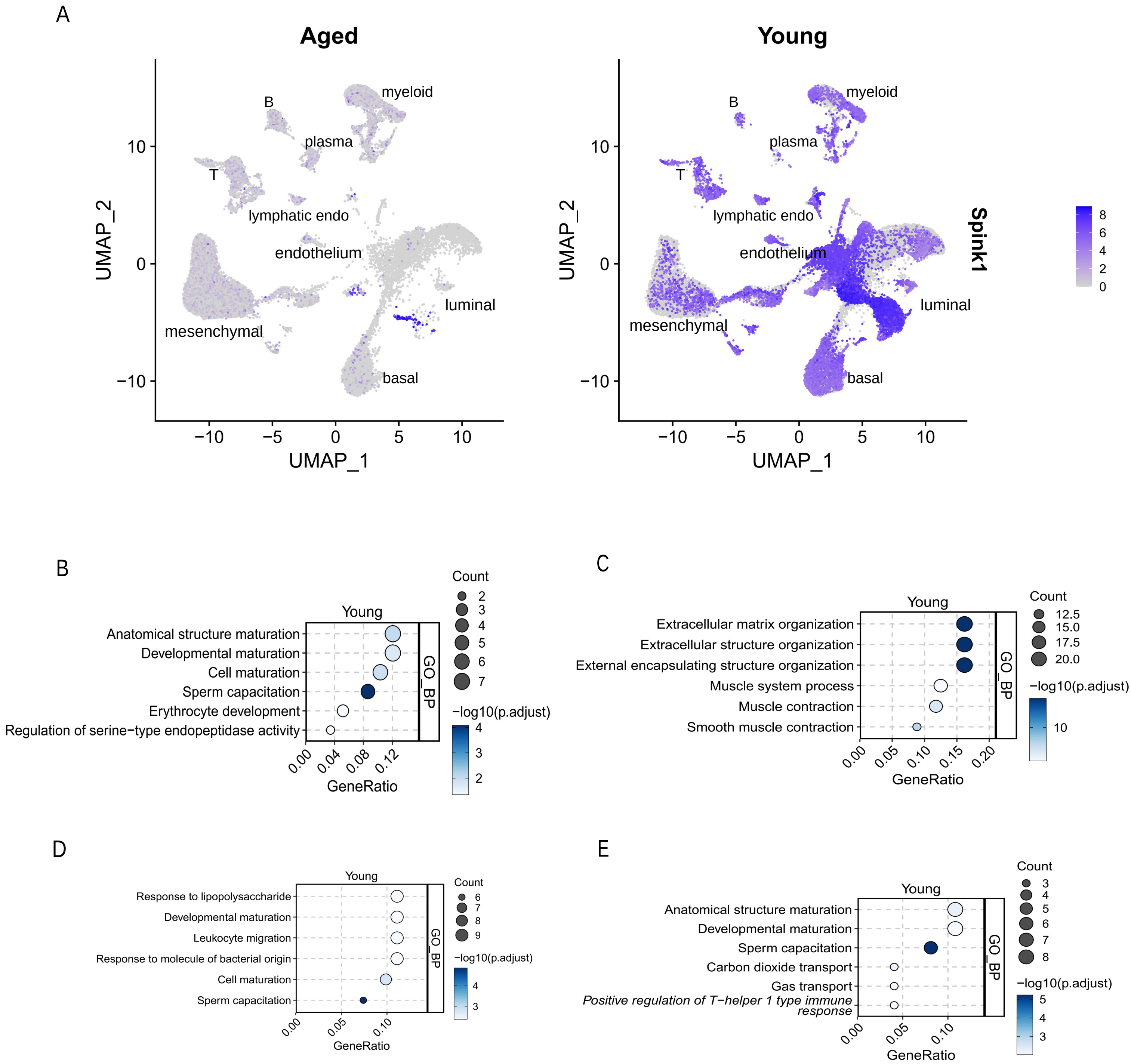

A
Aged
Young
myeloid
B
plasma
T
lymphatic endo
endothelium
mesenchymal
luminal
basal
UMAP_1
UMAP_2
Spink1
B
Young
Count
Anatomical structure maturation
Developmental maturation
Cell maturation
Sperm capacitation
Erythrocyte development
Regulation of serine−type endopeptidase activity
GO_BP
GeneRatio
−log10(p.adjust)
C
Young
Count
Extracellular matrix organization
Extracellular structure organization
External encapsulating structure organization
Muscle system process
Muscle contraction
Smooth muscle contraction
GO_BP
GeneRatio
−log10(p.adjust)
D
Young
Count
Response to lipopolysaccharide
Developmental maturation
Leukocyte migration
Response to molecule of bacterial origin
Cell maturation
Sperm capacitation
GO_BP
GeneRatio
−log10(p.adjust)
E
Young
Count
Anatomical structure maturation
Developmental maturation
Sperm capacitation
Carbon dioxide transport
Gas transport
Positive regulation of T−helper 1 type immune response
GO_BP
GeneRatio
−log10(p.adjust)

SFig4

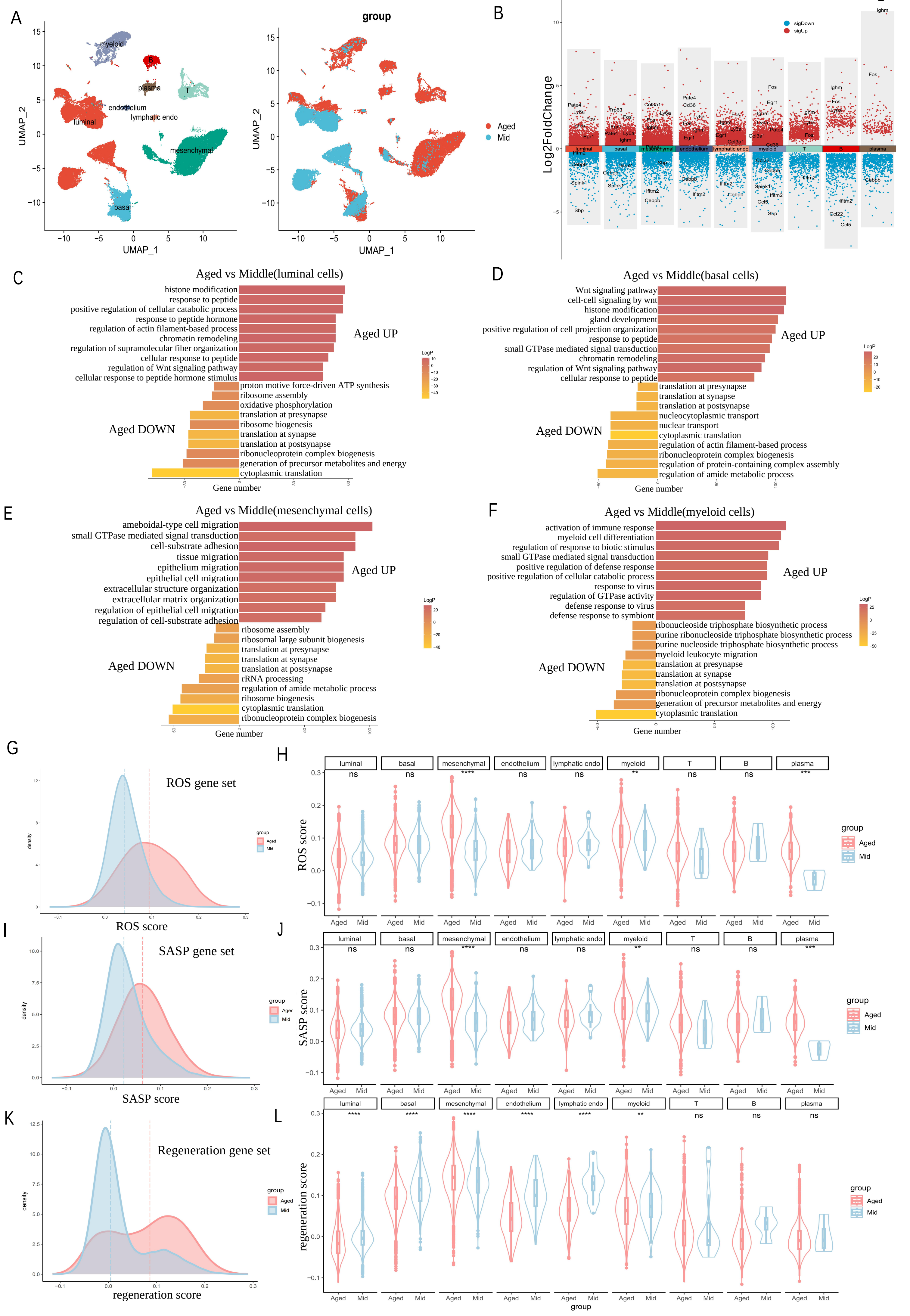
A
group
UMAP_1
UMAP_2
myeloid
B
plasma
T
endothelium
luminal
lymphatic endo
mesenchymal
basal
Aged
Mid
B
Log2FoldChange
sigDown
sigUp
luminal
basal
mesenchymal
endothelium
lymphatic endo
myeloid
T
B
plasma
C
Aged vs Middle(luminal cells)
histone modification
response to peptide
positive regulation of cellular catabolic process
response to peptide hormone
regulation of actin filament-based process
chromatin remodeling
regulation of supramolecular fiber organization
cellular response to peptide
regulation of Wnt signaling pathway
cellular response to peptide hormone stimulus
proton motive force-driven ATP synthesis
ribosome assembly
oxidative phosphorylation
translation at presynapse
ribosome biogenesis
translation at synapse
translation at postsynapse
ribonucleoprotein complex biogenesis
generation of precursor metabolites and energy
cytoplasmic translation
Aged UP
Aged DOWN
LogP
Gene number
D
Aged vs Middle(basal cells)
Wnt signaling pathway
cell-cell signaling by wnt
histone modification
gland development
positive regulation of cell projection organization
response to peptide
small GTPase mediated signal transduction
chromatin remodeling
regulation of Wnt signaling pathway
cellular response to peptide
translation at presynapse
translation at synapse
translation at postsynapse
nucleocytoplasmic transport
nuclear transport
cytoplasmic translation
regulation of actin filament-based process
ribonucleoprotein complex biogenesis
regulation of protein-containing complex assembly
regulation of amide metabolic process
Aged UP
Aged DOWN
LogP
Gene number
E
Aged vs Middle(mesenchymal cells)
ameboidal-type cell migration
small GTPase mediated signal transduction
cell-substrate adhesion
tissue migration
epithelium migration
epithelial cell migration
extracellular structure organization
extracellular matrix organization
regulation of epithelial cell migration
regulation of cell-substrate adhesion
ribosome assembly
ribosomal large subunit biogenesis
translation at presynapse
translation at synapse
translation at postsynapse
rRNA processing
regulation of amide metabolic process
ribosome biogenesis
cytoplasmic translation
ribonucleoprotein complex biogenesis
Aged UP
Aged DOWN
LogP
Gene number
F
Aged vs Middle(myeloid cells)
activation of immune response
myeloid cell differentiation
regulation of response to biotic stimulus
small GTPase mediated signal transduction
positive regulation of defense response
positive regulation of cellular catabolic process
response to virus
regulation of GTPase activity
defense response to virus
defense response to symbiont
ribonucleoside triphosphate biosynthetic process
purine ribonucleoside triphosphate biosynthetic process
purine nucleoside triphosphate biosynthetic process
myeloid leukocyte migration
translation at presynapse
translation at synapse
translation at postsynapse
ribonucleoprotein complex biogenesis
generation of precursor metabolites and energy
cytoplasmic translation
Aged UP
Aged DOWN
LogP
Gene number
G
ROS gene set
ROS score
density
H
ROS score
group
Aged
Mid
I
SASP gene set
SASP score
J
SASP score
K
Regeneration gene set
regeneration score
L
regeneration score
group

SFig5

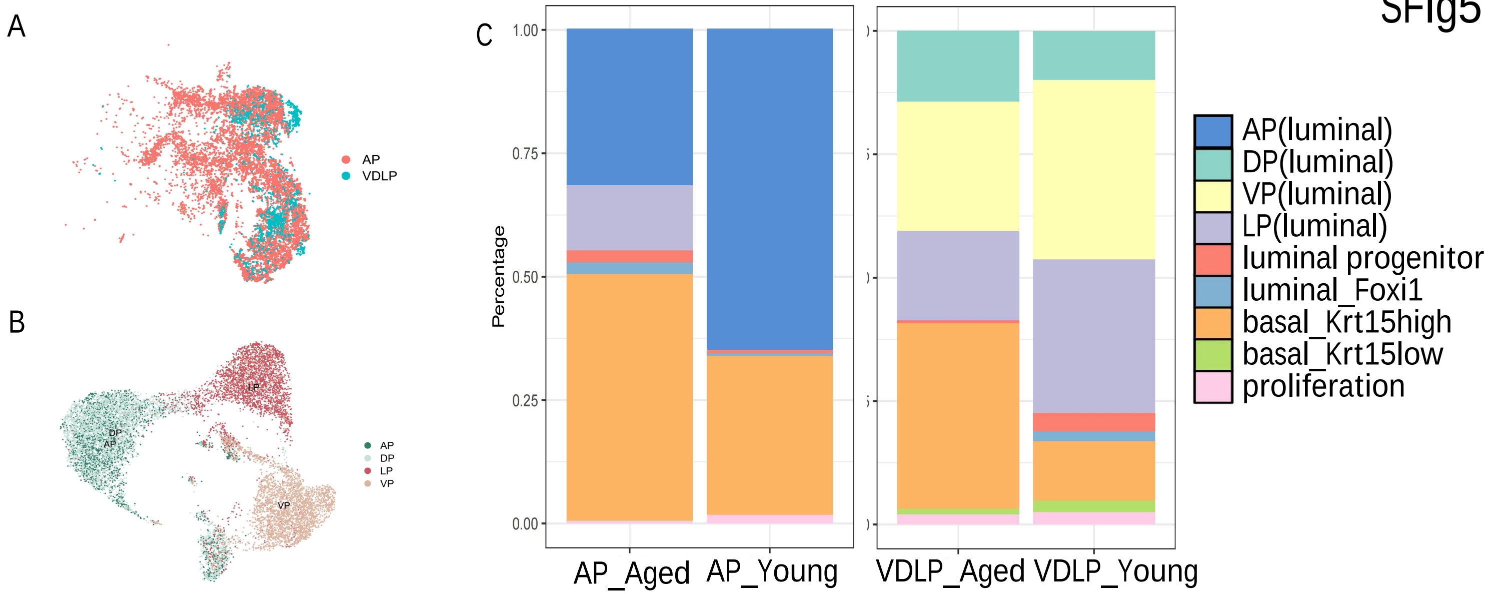
A
AP
VDLP
B
DP
AP
LP
VP
AP
DP
LP
VP
C
Percentage
1.00
0.75
0.50
0.25
0.00
AP_Aged
AP_Young
VDLP_Aged
VDLP_Young
AP(luminal)
DP(luminal)
VP(luminal)
LP(luminal)
luminal progenitor
luminal_Foxi1
basal_Krt15high
basal_Krt15low
proliferation

SFig6

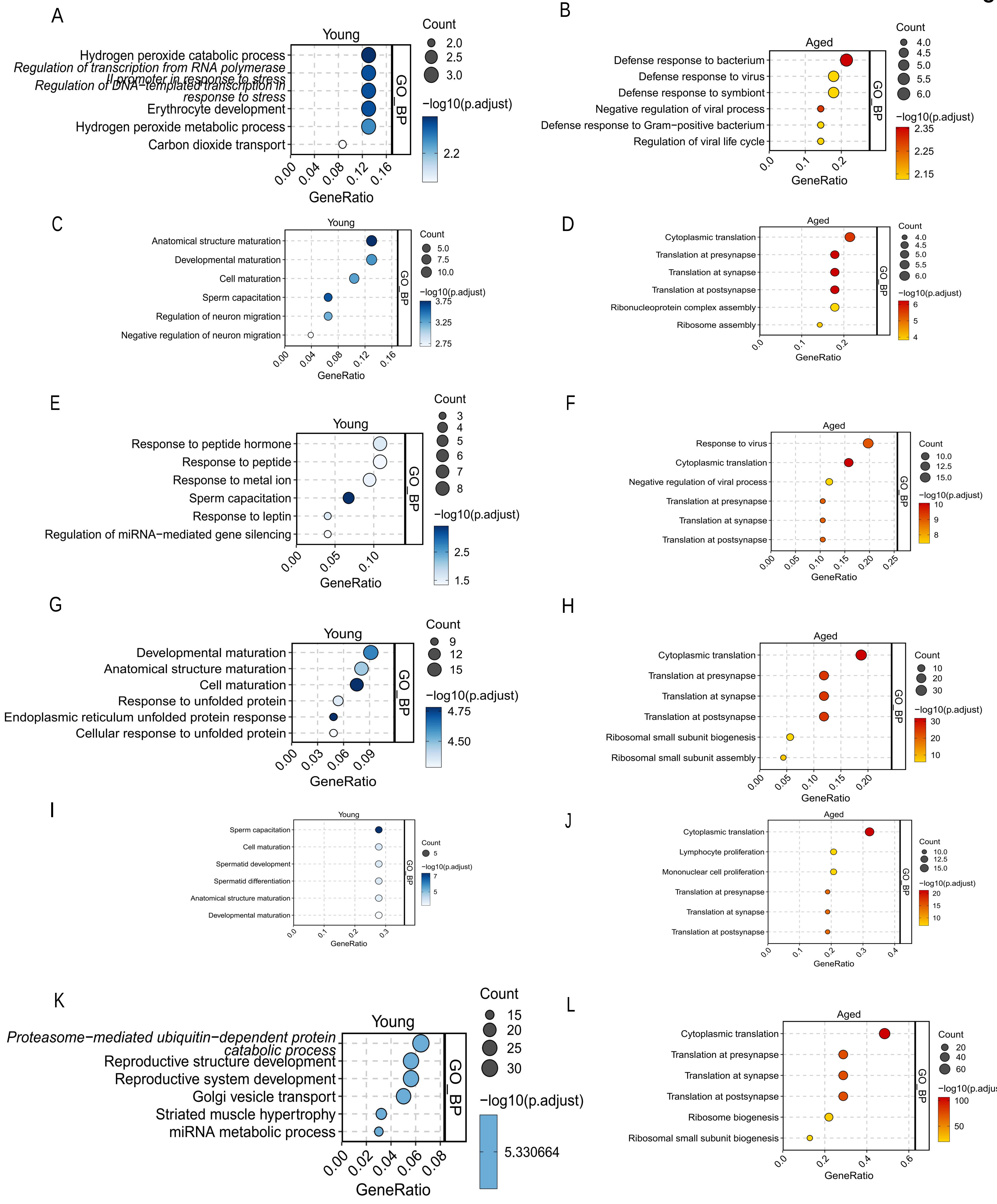
A
Young
Hydrogen peroxide catabolic process
Regulation of transcription from RNA polymerase II promoter in response to stress
Regulation of DNA−templated transcription in response to stress
Erythrocyte development
Hydrogen peroxide metabolic process
Carbon dioxide transport
GO_BP
GeneRatio
Count
−log10(p.adjust)
B
Aged
Defense response to bacterium
Defense response to virus
Defense response to symbiont
Negative regulation of viral process
Defense response to Gram−positive bacterium
Regulation of viral life cycle
C
Young
Anatomical structure maturation
Developmental maturation
Cell maturation
Sperm capacitation
Regulation of neuron migration
Negative regulation of neuron migration
D
Aged
Cytoplasmic translation
Translation at presynapse
Translation at synapse
Translation at postsynapse
Ribonucleoprotein complex assembly
Ribosome assembly
E
Young
Response to peptide hormone
Response to peptide
Response to metal ion
Sperm capacitation
Response to leptin
Regulation of miRNA−mediated gene silencing
F
Aged
Response to virus
Cytoplasmic translation
Negative regulation of viral process
Translation at presynapse
Translation at synapse
Translation at postsynapse
G
Young
Developmental maturation
Anatomical structure maturation
Cell maturation
Response to unfolded protein
Endoplasmic reticulum unfolded protein response
Cellular response to unfolded protein
H
Aged
Cytoplasmic translation
Translation at presynapse
Translation at synapse
Translation at postsynapse
Ribosomal small subunit biogenesis
Ribosomal small subunit assembly
I
Young
Sperm capacitation
Cell maturation
Spermatid development
Spermatid differentiation
Anatomical structure maturation
Developmental maturation
J
Aged
Cytoplasmic translation
Lymphocyte proliferation
Mononuclear cell proliferation
Translation at presynapse
Translation at synapse
Translation at postsynapse
K
Young
Proteasome−mediated ubiquitin−dependent protein catabolic process
Reproductive structure development
Reproductive system development
Golgi vesicle transport
Striated muscle hypertrophy
miRNA metabolic process
5.330664
L
Aged
Cytoplasmic translation
Translation at presynapse
Translation at synapse
Translation at postsynapse
Ribosome biogenesis
Ribosomal small subunit biogenesis

SFig7

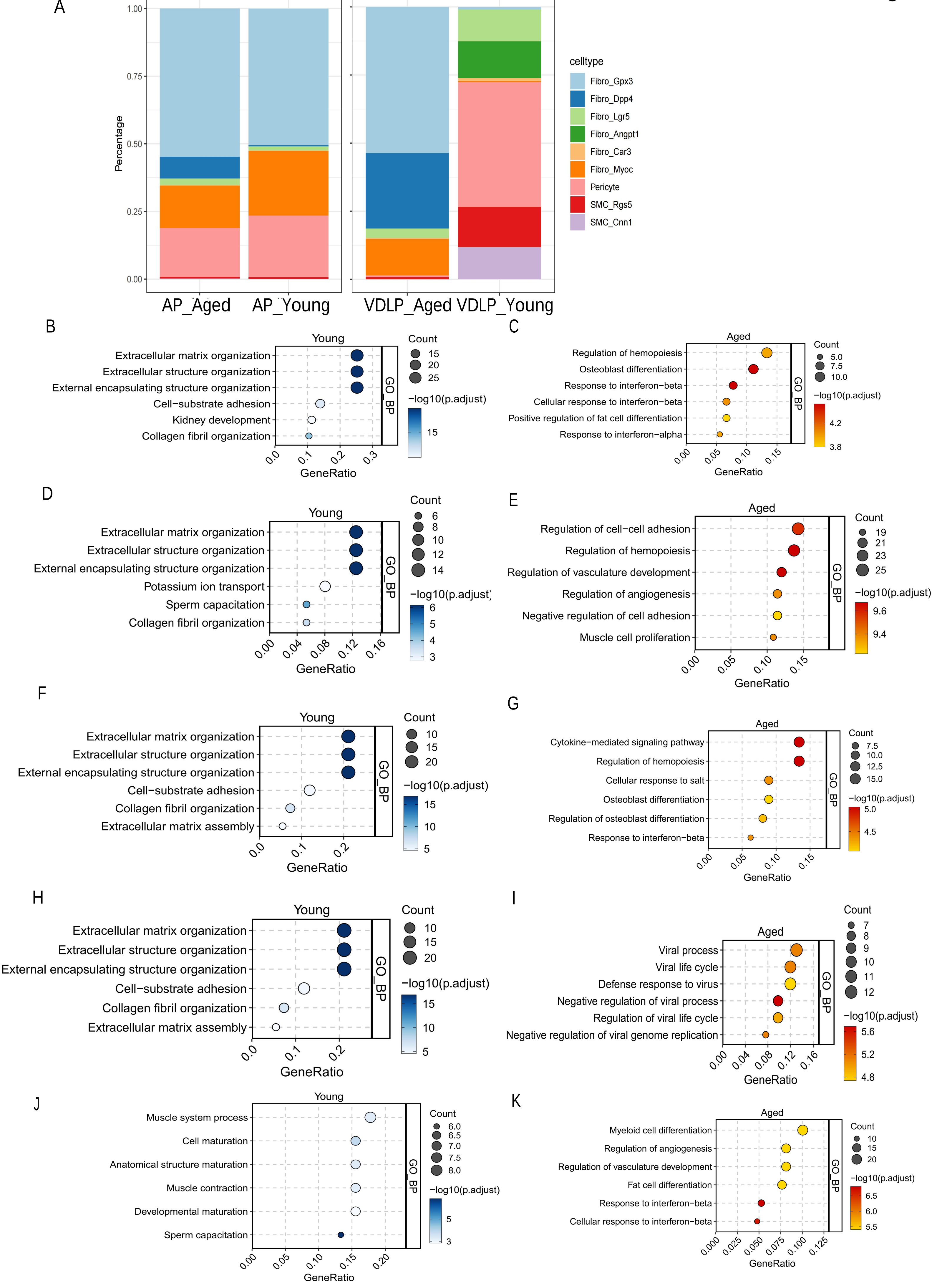
A
Percentage
1.00
0.75
0.50
0.25
0.00
AP_Aged
AP_Young
VDLP_Aged
VDLP_Young
celltype
Fibro_Gpx3
Fibro_Dpp4
Fibro_Lgr5
Fibro_Angpt1
Fibro_Car3
Fibro_Myoc
Pericyte
SMC_Rgs5
SMC_Cnn1
B
Young
Extracellular matrix organization
Extracellular structure organization
External encapsulating structure organization
Cell−substrate adhesion
Kidney development
Collagen fibril organization
GO_BP
GeneRatio
0.0 0.1 0.2 0.3
Count
15
20
25
−log10(p.adjust)
15
C
Aged
Regulation of hemopoiesis
Osteoblast differentiation
Response to interferon−beta
Cellular response to interferon−beta
Positive regulation of fat cell differentiation
Response to interferon−alpha
GO_BP
GeneRatio
0.00 0.05 0.10 0.15
Count
5.0
7.5
10.0
−log10(p.adjust)
4.2
3.8
D
Young
Extracellular matrix organization
Extracellular structure organization
External encapsulating structure organization
Potassium ion transport
Sperm capacitation
Collagen fibril organization
GO_BP
GeneRatio
0.00 0.04 0.08 0.12 0.16
Count
6
8
10
12
14
−log10(p.adjust)
6
5
4
3
E
Aged
Regulation of cell−cell adhesion
Regulation of hemopoiesis
Regulation of vasculature development
Regulation of angiogenesis
Negative regulation of cell adhesion
Muscle cell proliferation
GO_BP
GeneRatio
0.00 0.05 0.10 0.15
Count
19
21
23
25
−log10(p.adjust)
9.6
9.4
F
Young
Extracellular matrix organization
Extracellular structure organization
External encapsulating structure organization
Cell−substrate adhesion
Collagen fibril organization
Extracellular matrix assembly
GO_BP
GeneRatio
0.0 0.1 0.2
Count
10
15
20
−log10(p.adjust)
15
10
5
G
Aged
Cytokine−mediated signaling pathway
Regulation of hemopoiesis
Cellular response to salt
Osteoblast differentiation
Regulation of osteoblast differentiation
Response to interferon−beta
GO_BP
GeneRatio
0.00 0.05 0.10 0.15
Count
7.5
10.0
12.5
15.0
−log10(p.adjust)
5.0
4.5
H
Young
Extracellular matrix organization
Extracellular structure organization
External encapsulating structure organization
Cell−substrate adhesion
Collagen fibril organization
Extracellular matrix assembly
GO_BP
GeneRatio
0.0 0.1 0.2
Count
10
15
20
−log10(p.adjust)
15
10
5
I
Aged
Viral process
Viral life cycle
Defense response to virus
Negative regulation of viral process
Regulation of viral life cycle
Negative regulation of viral genome replication
GO_BP
GeneRatio
0.00 0.04 0.08 0.12 0.16
Count
7
8
9
10
11
12
−log10(p.adjust)
5.6
5.2
4.8
J
Young
Muscle system process
Cell maturation
Anatomical structure maturation
Muscle contraction
Developmental maturation
Sperm capacitation
GO_BP
GeneRatio
0.00 0.05 0.10 0.15 0.20
Count
6.0
6.5
7.0
7.5
8.0
−log10(p.adjust)
5
3
K
Aged
Myeloid cell differentiation
Regulation of angiogenesis
Regulation of vasculature development
Fat cell differentiation
Response to interferon−beta
Cellular response to interferon−beta
GO_BP
GeneRatio
0.000 0.025 0.050 0.075 0.100 0.125
Count
10
15
20
−log10(p.adjust)
6.5
6.0
5.5

SFig8

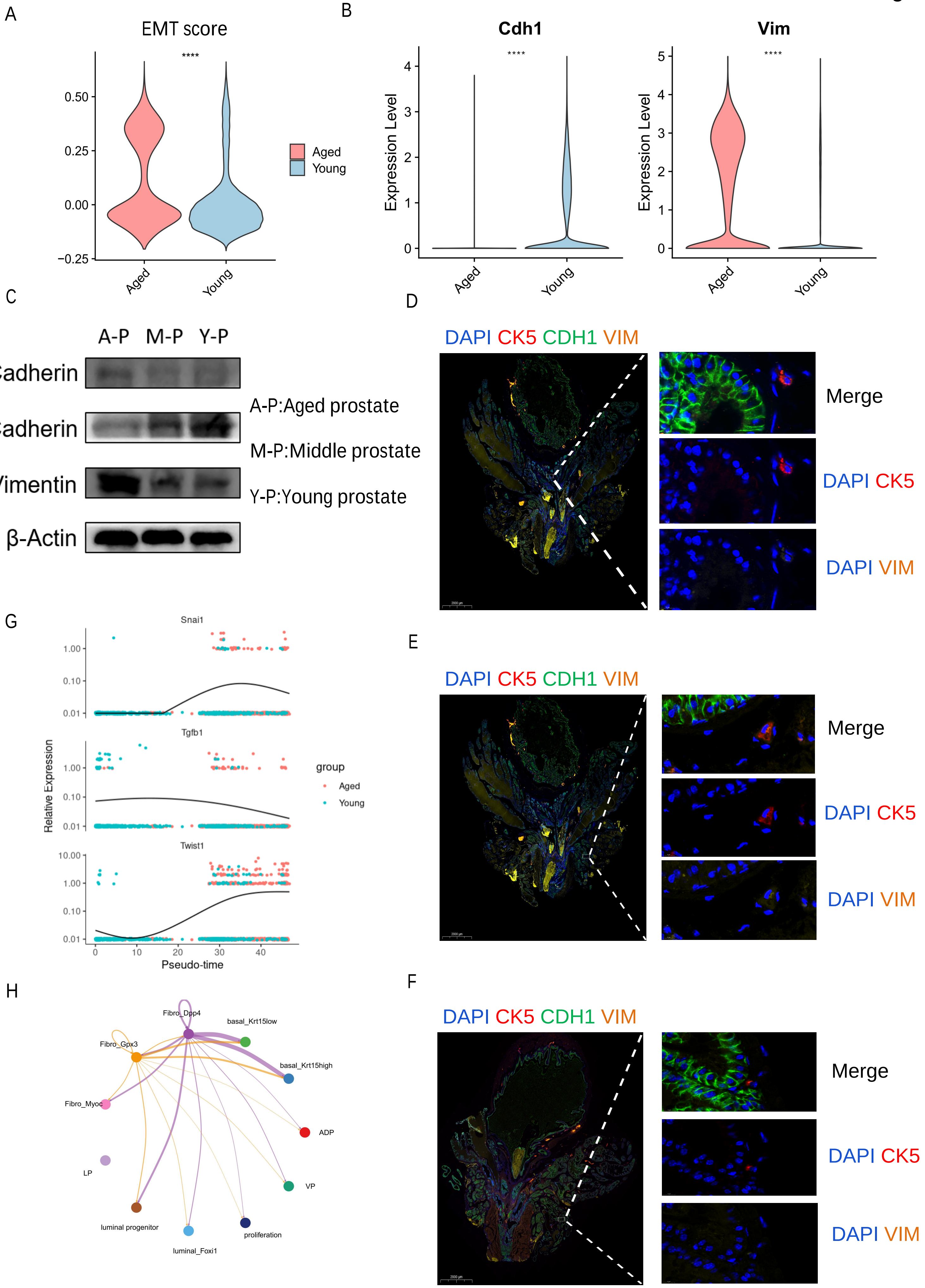
A
EMT score
****
0.50
0.25
0.00
−0.25
Aged
Young
B
Cdh1
Vim
Expression Level
C
A-P M-P Y-P
N-Cadherin
E-Cadherin
Vimentin
β-Actin
A-P:Aged prostate
M-P:Middle prostate
Y-P:Young prostate
D
DAPI CK5 CDH1 VIM
Merge
DAPI CK5
DAPI VIM
E
DAPI CK5 CDH1 VIM
Merge
DAPI CK5
DAPI VIM
F
DAPI CK5 CDH1 VIM
Merge
DAPI CK5
DAPI VIM
G
Snai1
Tgfb1
Twist1
Relative Expression
group
Aged
Young
Pseudo-time
H
Fibro_Dpp4
basal_Krt15low
Fibro_Gpx3
basal_Krt15high
Fibro_Myoc
ADP
LP
VP
luminal progenitor
luminal_Foxi1
proliferation

SFig9

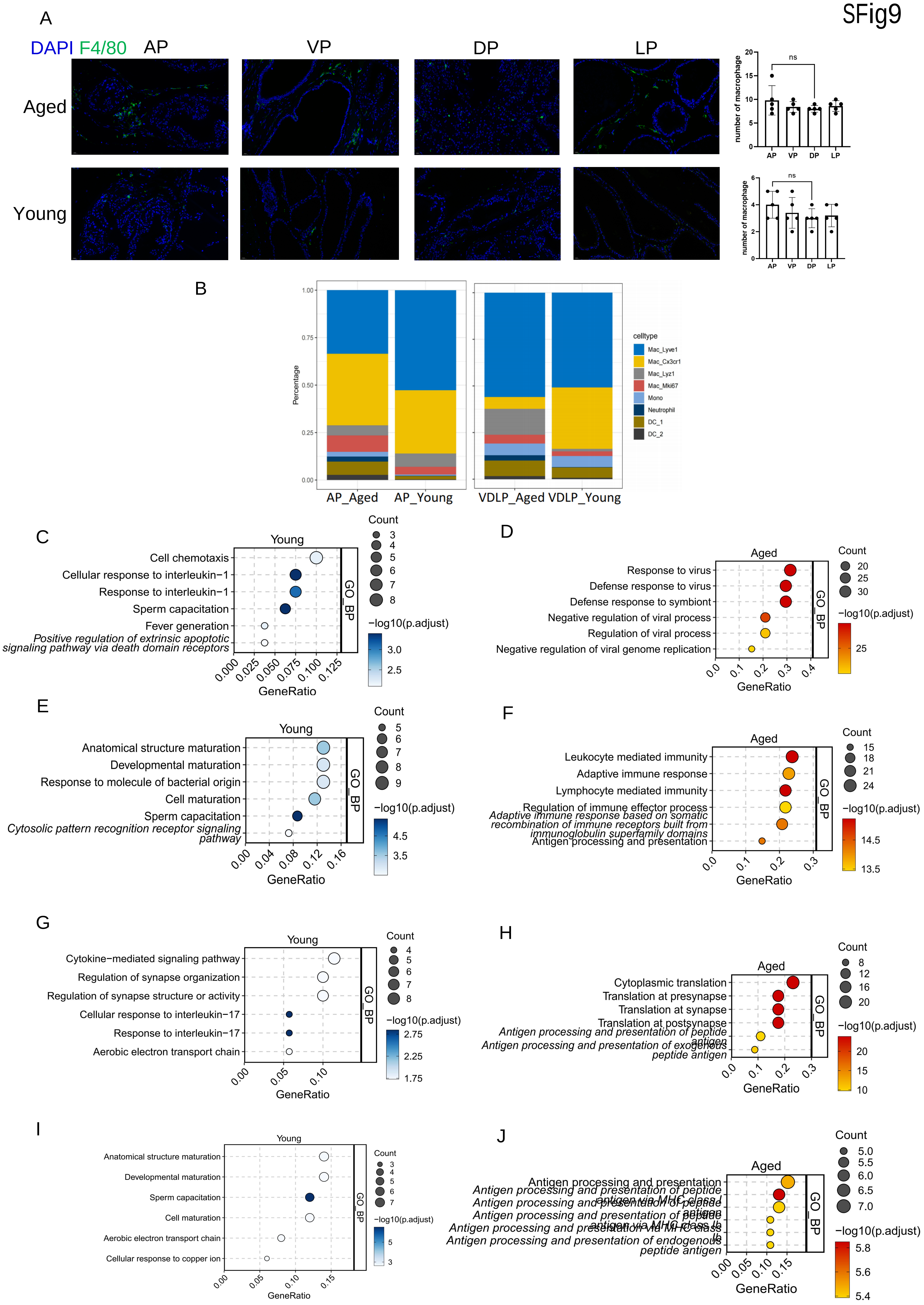

A
DAPI F4/80
AP
VP
DP
LP
Aged
Young
number of macrophage
ns
AP VP DP LP
B
1.00
0.75
0.50
0.25
0.00
Percentage
AP_Aged
AP_Young
VDLP_Aged
VDLP_Young
celltype
Mac_Lyve1
Mac_Cx3cr1
Mac_Lyz1
Mac_Mki67
Mono
Neutrophil
DC_1
DC_2
C
Young
Cell chemotaxis
Cellular response to interleukin−1
Response to interleukin−1
Sperm capacitation
Fever generation
Positive regulation of extrinsic apoptotic signaling pathway via death domain receptors
GO_BP
GeneRatio
Count
−log10(p.adjust)
D
Aged
Response to virus
Defense response to virus
Defense response to symbiont
Negative regulation of viral process
Regulation of viral process
Negative regulation of viral genome replication
GO_BP
GeneRatio
Count
−log10(p.adjust)
E
Young
Anatomical structure maturation
Developmental maturation
Response to molecule of bacterial origin
Cell maturation
Sperm capacitation
Cytosolic pattern recognition receptor signaling pathway
GO_BP
GeneRatio
Count
−log10(p.adjust)
F
Aged
Leukocyte mediated immunity
Adaptive immune response
Lymphocyte mediated immunity
Regulation of immune effector process
Adaptive immune response based on somatic recombination of immune receptors built from immunoglobulin superfamily domains
Antigen processing and presentation
GO_BP
GeneRatio
Count
−log10(p.adjust)
G
Young
Cytokine−mediated signaling pathway
Regulation of synapse organization
Regulation of synapse structure or activity
Cellular response to interleukin−17
Response to interleukin−17
Aerobic electron transport chain
GO_BP
GeneRatio
Count
−log10(p.adjust)
H
Aged
Cytoplasmic translation
Translation at presynapse
Translation at synapse
Translation at postsynapse
Antigen processing and presentation of peptide antigen
Antigen processing and presentation of exogenous peptide antigen
GO_BP
GeneRatio
Count
−log10(p.adjust)
I
Young
Anatomical structure maturation
Developmental maturation
Sperm capacitation
Cell maturation
Aerobic electron transport chain
Cellular response to copper ion
GO_BP
GeneRatio
Count
−log10(p.adjust)
J
Aged
Antigen processing and presentation
Antigen processing and presentation of peptide antigen via MHC class I
Antigen processing and presentation of peptide antigen
Antigen processing and presentation of peptide antigen via MHC class Ib
Antigen processing and presentation via MHC class Ib
Antigen processing and presentation of endogenous peptide antigen
GO_BP
GeneRatio
Count
−log10(p.adjust)

SFig10

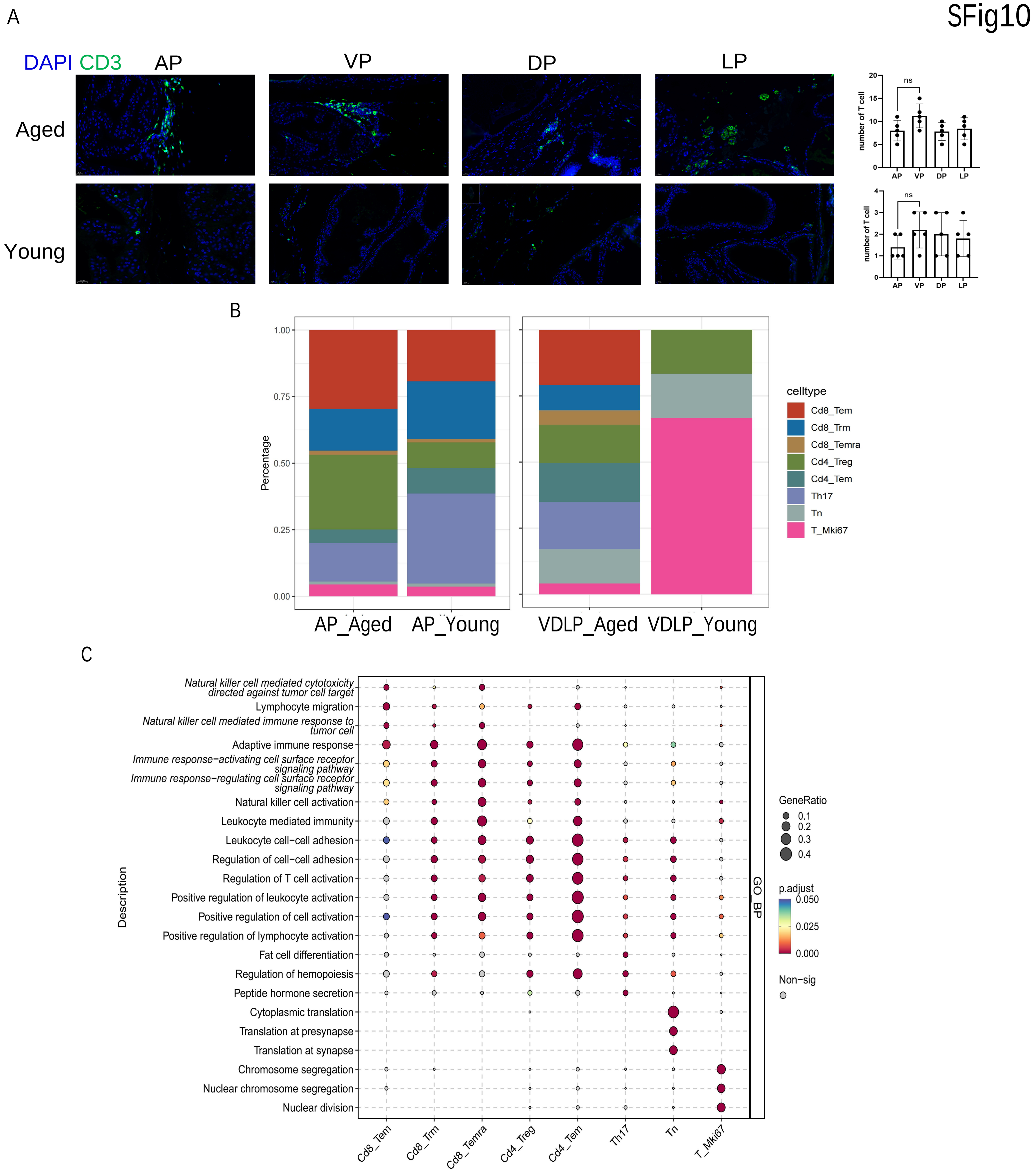
A
DAPI CD3
AP
VP
DP
LP
Aged
Young
number of T cell
ns
AP
VP
DP
LP
B
Percentage
AP_Aged
AP_Young
VDLP_Aged
VDLP_Young
celltype
Cd8_Tem
Cd8_Trm
Cd8_Temra
Cd4_Treg
Cd4_Tem
Th17
Tn
T_Mki67
C
Description
Natural killer cell mediated cytotoxicity directed against tumor cell target
Lymphocyte migration
Natural killer cell mediated immune response to tumor cell
Adaptive immune response
Immune response−activating cell surface receptor signaling pathway
Immune response−regulating cell surface receptor signaling pathway
Natural killer cell activation
Leukocyte mediated immunity
Leukocyte cell−cell adhesion
Regulation of cell−cell adhesion
Regulation of T cell activation
Positive regulation of leukocyte activation
Positive regulation of cell activation
Positive regulation of lymphocyte activation
Fat cell differentiation
Regulation of hemopoiesis
Peptide hormone secretion
Cytoplasmic translation
Translation at presynapse
Translation at synapse
Chromosome segregation
Nuclear chromosome segregation
Nuclear division
GO_BP
GeneRatio
p.adjust
Non−sig
Cd8_Tem
Cd8_Trm
Cd8_Temra
Cd4_Treg
Cd4_Tem
Th17
Tn
T_Mki67
